# A Morphing [4Fe‐3S‐nO]‐Cluster within a Carbon Monoxide Dehydrogenase Scaffold

**DOI:** 10.1002/anie.202117000

**Published:** 2022-03-04

**Authors:** Jae‐Hun Jeoung, Jochen Fesseler, Lilith Domnik, Friederike Klemke, Malte Sinnreich, Christian Teutloff, Holger Dobbek

**Affiliations:** ^1^ Humboldt-Universität zu Berlin Institut für Biologie Unter den Linden 6 10099 Berlin Germany; ^2^ Freie Universität Berlin, Fachbereich Physik Arnimallee 14 14195 Berlin Germany

**Keywords:** CODH Evolution, Enzymes, Hydroxylamine Reduction, Iron-Sulfur-Oxo Cluster, Structural Biology

## Abstract

Ni,Fe‐containing carbon monoxide dehydrogenases (CODHs) catalyze the reversible reduction of CO_2_ to CO. Several anaerobic microorganisms encode multiple CODHs in their genome, of which some, despite being annotated as CODHs, lack a cysteine of the canonical binding motif for the active site Ni,Fe‐cluster. Here, we report on the structure and reactivity of such a deviant enzyme, termed CooS‐V_Ch_. Its structure reveals the typical CODH scaffold, but contains an iron‐sulfur‐oxo hybrid‐cluster. Although closely related to true CODHs, CooS‐V_Ch_ catalyzes neither CO oxidation, nor CO_2_ reduction. The active site of CooS‐V_Ch_ undergoes a redox‐dependent restructuring between a reduced [4Fe‐3S]‐cluster and an oxidized [4Fe‐2S‐S*‐2O‐2(H_2_O)]‐cluster. Hydroxylamine, a slow‐turnover substrate of CooS‐V_Ch_, oxidizes the hybrid‐cluster in two structurally distinct steps. Overall, minor changes in CODHs are sufficient to accommodate a Fe/S/O‐cluster in place of the Ni,Fe‐heterocubane‐cluster of CODHs.

## Graphical abstract

The exchange of one amino acid (Cys‐>Glu) coordinating the Ni/Fe/S‐cluster in carbon monoxide dehydrogenases (CODH) is the main difference resulting in the formation of a Fe/S/O‐hybrid‐cluster, found in CooS‐V, while conserving the overall architecture of CODHs. The Fe/S/O‐hybrid‐cluster in CooS‐V reversibly rearranges dependent on its redox‐state.

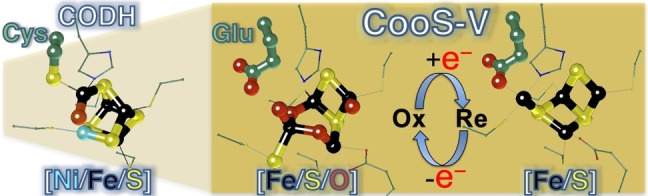

## Introduction

Ni,Fe‐containing carbon monoxide dehydrogenases (CODHs) catalyze the reversible two‐electron reduction of CO_2_ to CO and water (CO_2_+2 H^+^+2 e^−^↔CO+H_2_O). They are employed in anaerobic bacteria and archaea, including acetogenic bacteria, sulfate‐reducing bacteria and archaea, hydrogenogenic bacteria and methanogenic archaea for different physiological roles. Their typical functions are either to gain reducing equivalents by oxidizing CO to CO_2_ or to reduce CO_2_ to CO for further reactions, e.g. to synthesize acetyl‐CoA in the bifunctional complex between a CODH and a Ni,Ni‐containing acetyl‐coenzyme A synthase.[^1^, ^2^] Based on overall structures, the smaller CooS‐type CODHs and the larger Cdh‐type CODHs can be distinguished.

The structures of different types of CODHs have been reported.[^3^, ^4^, ^5^, ^6^, ^7^, ^8^, ^9^, ^10^, ^11^, ^12^] CODHs form dimers containing up to seven [4Fe‐4S]‐clusters for electron transfer and two [Ni4Fe‐4S]‐clusters for catalysis. The unique [Ni4Fe‐4S]‐cluster is harbored in the active site of CODHs, where it is coordinated by a set of conserved residues: five cysteines and one histidine.

Phylogenetic analysis and biogeochemical data imply that CODHs belong to the set of enzymes with which the last universal common ancestor was equipped.[^13^, ^14^] According to sequence and structural similarity, CODHs share a common ancestor with the hybrid‐cluster proteins (HCPs) giving rise to the prismane/CODH family (Pfam: PF03063).^[15]^ Members of the prismane/CODH family share several structural motifs, including overlapping coordination motifs for iron–sulfur clusters.

Like CODHs, HCPs are widely distributed among (facultative) anaerobic microorganisms, including bacteria, archaea, and unicellular eukaryotes, of which several HCPs have been characterized.[^16^, ^17^, ^18^, ^19^] Unlike CODHs, HCPs are unable to catalyze CO‐oxidation,^[20]^ but support the reduction of hydroxylamine, peroxide and NO and the formation of S‐nitrosothiols.[^20^, ^21^, ^22^, ^23^] The mechanistic basis for both reactivities, e.g. place of substrate binding and the role of the hybrid‐cluster in these reactions is unknown.^[20]^

**Figure 1:**
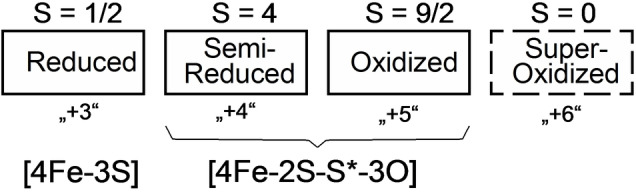
Four oxidation states of the hybrid‐cluster of HCPs. The number in parenthesis are not giving the charge, but refer to an older naming convention. Boxes with solid lines indicate oxidation states of likely physiological relevance.

HCPs have been extensively characterized using spectroscopic approaches,[^17^, ^18^, ^19^, ^20^, ^24^, ^25^] as well as by protein crystallography.[^16^, ^26^, ^27^, ^28^, ^29^] Structurally characterized HCPs carry two metal clusters: a [4Fe‐4S]‐ or [2Fe‐2S]‐cluster and the hybrid‐cluster. The hybrid‐cluster is a unique Fe/S/O‐cluster being a hybrid of an iron‐sulfur‐ and an iron‐oxo‐cluster. The composition of the hybrid‐cluster depends on its oxidation state. The as‐isolated, oxidized state, may be described as a [4Fe‐2S‐S*‐3O]‐cluster containing two μ‐S, three μ‐O bridges and a cysteine persulfide (S*).[^16^, ^20^, ^27^, ^28^] Upon reduction, the [4Fe‐2S‐S*‐3O]‐cluster is transformed into a [4Fe‐3S]‐cluster without μ‐O bridges, whereby the persulfido sulfur becomes a sulfido‐ligand of the cluster.[^29^, ^30^] The hybrid‐cluster may attain four different oxidation states (Figure 1).^[27]^

An HCP and several CODHs are encoded in *Carboxydothermus hydrogenoformans*, a hydrogenogenic bacterium able to grow lithoautotrophically on CO as sole carbon source.^[31]^ Its genome contains five *cooS* genes (*cooS*‐I to *cooS*‐V) which have been annotated to encode CODHs.^[32]^ Four *cooS* genes are found in gene clusters hinting to their role in CO metabolism and their products, CODH‐I to ‐IV, catalyze the reversible CO oxidation under different physiological conditions.[^31^, ^32^, ^33^, ^34^]

In contrast, the gene product of CHY_RS00160, annotated as CODH‐V, (termed CooS‐V_*Ch*_) has not been investigated until now. Phylogenetic analysis places the CooS‐V_*Ch*_ gene together with a variety of other uncharacterized CODHs, whose genomic contexts lack obvious links to C1‐metabolism and are only found in organisms with more than one gene encoding a CODH, including several bacteria isolated from the termite gut.^[35]^

Here, we analyzed the structure and biochemical properties of CooS‐V_*Ch*_ and outline its potential route of evolution from a CODH progenitor.

## Results and Discussion

### HCP, CooS‐ and Cdh‐Type CODHs

**Figure 2:**
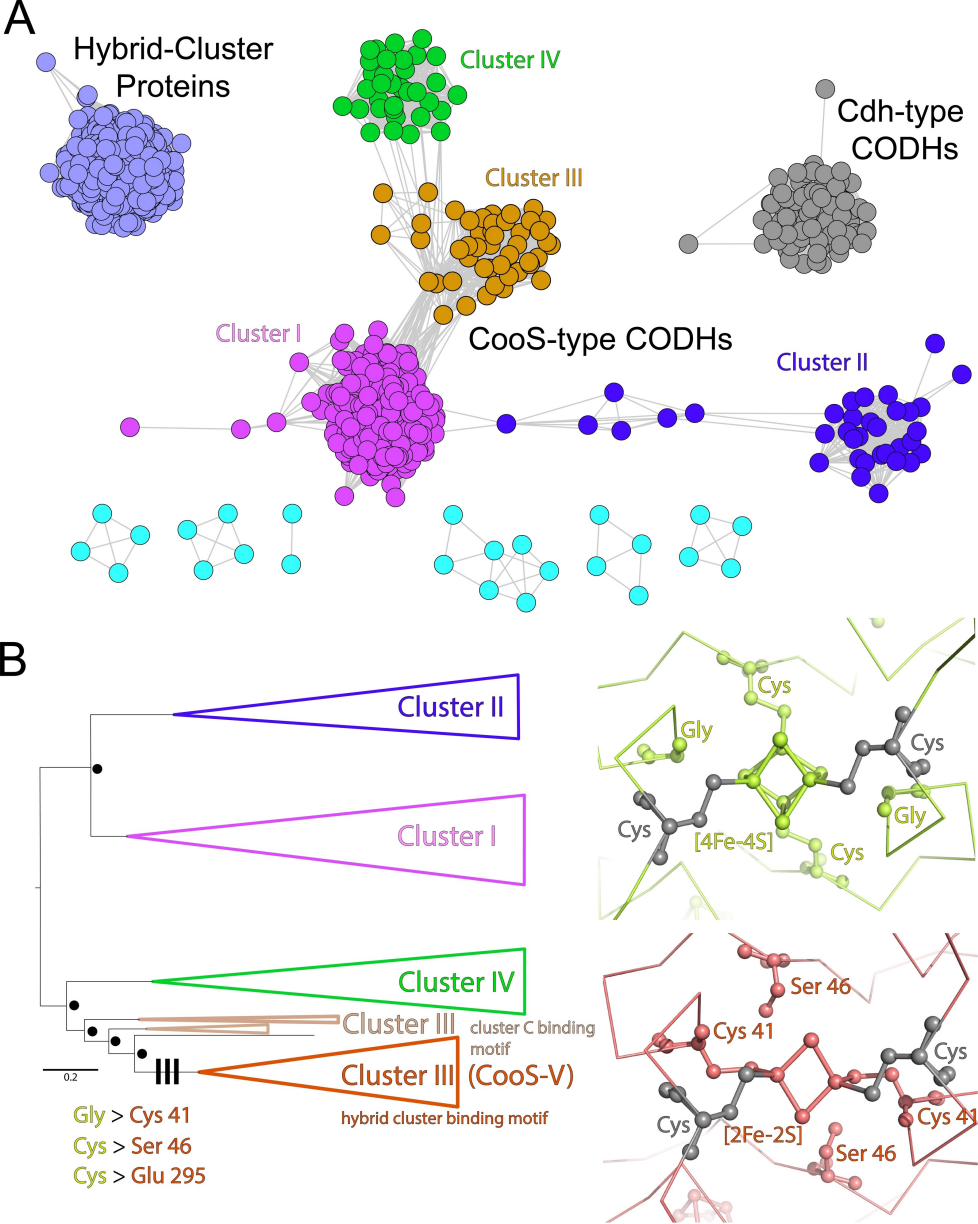
Sequence similarity and phylogenetic analysis. A) A SSN containing selected sequences of InterPro entry IPR004137 (HCP/CODH enzyme family). B) Simplified phylogeny of CooS‐type CODHs, colored as in A. The complete phylogeny can be found in Figure S1. Dots mark nodes with a Bayesian‐like transformation of approximate likelihood ratio test support of 1. Lines mark the trait evolution at three selected sequence positions. The figures on the right show the structural evolution for the two exchanges near the bridging cluster. The ancestral state in green, modeled using the structure of CODH‐II_*Ch*_ (PDB‐ID: 4UDY^[36]^), contains the ancestral traits at this position. The derived amino acids are colored red‐brown, using the structure of CooS‐V_*Ch*_ as a model. Cysteine residues shown in gray are conserved and complete the coordination of the bridging Fe/S‐clusters.

We used InterPro entry IPR004137 containing originally 9634 sequences, from which similar sequences were removed to give 1270 sequences to be analyzed in a sequence similarity network (SSN, Figure 2A). At the chosen level of similarity, HCPs and Cdh‐type CODHs are clearly separated. Also, the CooS‐type CODHs are separate, but fall into four clusters (clusters I–IV). Cluster I is largest and contains the monofunctional CODHs from *Rhodospirillum rubrum*, *C. hydrogenoformans* (CODH‐I, CODH‐II and CODH‐IV), *Desulfovibrio vulgaris* as well as the bifunctional CODH from *Moorella thermoacetica*. Cluster III contains sequences of archaeal and bacterial sources including CooS‐V_*Ch*_, which are annotated as CODHs.

A phylogeny of 384 selected sequences of CooS‐type CODHs, including members of all four clusters, shows that the clusters identified in the SSN also form distinct clades in the phylogenic tree (Figure S1). A clade containing the sequences of cluster III harbors a subclade distinguished by two exchanges of iron‐sulfur cluster coordinating residues (Figure 2B), particularly the exchange of a cluster C coordinating cysteine residue (Cys295) to glutamate. In this subclade the sequence of CooS‐V_*Ch*_ is found.

### Biochemical Investigation of CooS‐V_Ch_

CooS‐V_*Ch*_ was heterologously expressed in *E. coli*, using the growth conditions previously described to produce recombinant CODH‐II_*Ch*_.^[5]^ CooS‐V_*Ch*_ was isolated by one‐step affinity chromatography, yielding a homogeneous preparation with a unique, symmetrical peak in an analytical size exclusion chromatogram, corresponding to a dimer with an apparent molecular weight of 118 kDa (Figure S2‐A).

The isolated protein had a reddish‐brown color and displayed the typical UV/Vis spectral features of an Fe/S‐cluster containing protein with peaks at 278 and 405 nm and shoulders at 325, 455 and 505 nm (Figure S2‐B). The individual maxima have extinction coefficients of 112 mM^−1^ cm^−1^ (*ϵ*_280_), 41.6 mM^−1^ cm^−1^ (*ϵ*_325_), 34.5 mM^−1^ cm^−1^ (*ϵ*_405_), 25.6 mM^−1^ cm^−1^ (*ϵ*_455_) and 15.6 mM^−1^ cm^−1^ (*ϵ*_505_). Reductive titration with Na‐dithionite (DT, Figure S2‐B arrow) decreased absorption in the region of 300–600 nm. The most notable change of absorption upon reduction was observed at 405 nm (Δ*ϵ*=14 mM^−1^ cm^−1^, Figure S2‐B inset).

CooS‐V_*Ch*_ was tested for different reactivities. Based on its annotation as a CODH, we initially tested if CooS‐V_*Ch*_ catalyzes CO‐oxidation or CO_2_‐reduction, but both could not be detected. We also tested CooS‐V_*Ch*_ ability to reduce NO and hydroxylamine (NH_2_OH). To assay NO reductase activity, we used two different methods of detection and two different ways of supplying reducing equivalents (see details in Supporting Experimental Procedures). Despite the different conditions employed, we did not detect catalytic NO reduction by CooS‐V_*Ch*_ above the high background reactivity of NO. The two‐electron reduction of NH_2_OH to ammonia and water (NH_2_OH+2 e^−^+2 H^+^→NH_3_+H_2_O) has been described for both CODHs and HCPs.[^19^, ^37^, ^38^, ^39^] We determined the NH_2_OH reductase activity in a concentration range between 0 and 100 mM NH_2_OH with reduced methyl viologen as artificial electron donor. The specific activity was determined from reoxidation of the reduced methyl viologen and exhibited a hyperbolic dependency on the NH_2_OH concentration (Figure S2‐C). The observed NH_2_OH reductase activity is very low with a maximal turnover rate *k*_cat_ of 0.13 s^−1^ and a resulting catalytic efficiency of *k*_cat_/*K*_M_ of 20 M^−1^ s^−1^. HCPs vary in their reactivity with NH_2_OH, but have typically two orders of magnitude higher catalytic efficiency than CooS‐V_*Ch*_. Even the monofunctional CODH from *R. rubrum* has a more than 20‐fold higher *k*_cat_/*K*_M_ value (450 M^−1^ s^−1^) for turnover of NH_2_OH, which is further increased to 1600 M^−1^ s^−1^ by exchanging the cluster C coordinating histidine residue (H265V).^[39]^

### Electron Paramagnetic Resonance (EPR) Spectroscopy

**Figure 3:**
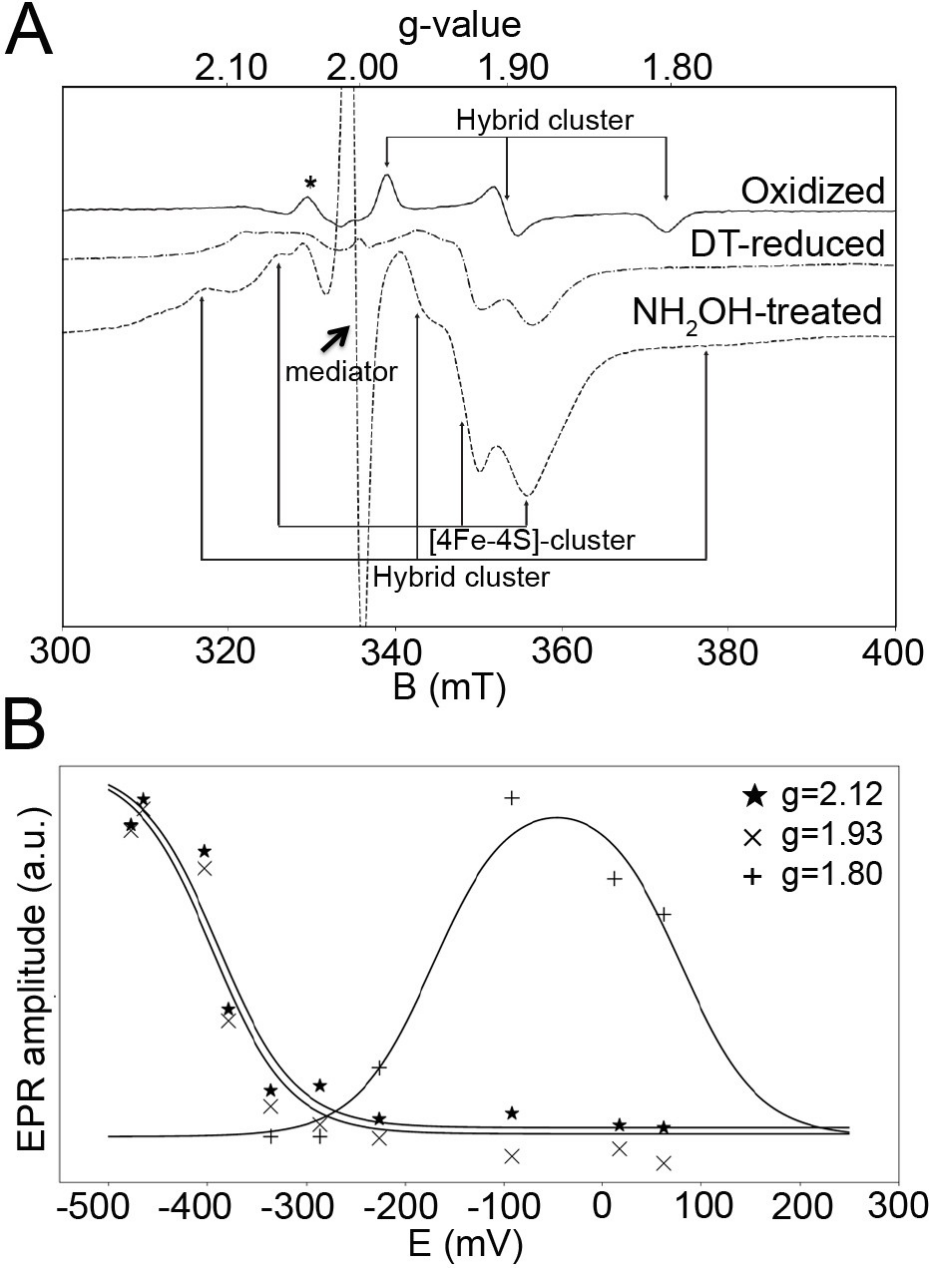
EPR spectroscopic analysis of CooS‐V_*Ch*_. A) EPR of CooS‐V_*Ch*_ at 10 K, assigned species are indicated, *: redox‐potential independent signal of an assumingly broken Fe/S‐cluster, experiments were performed at f_MW_=9.4 GHz, microwave power 50 μW, 10 G modulation amplitude. B) EPR‐monitored redox titration of CooS‐V_*Ch*_ and Nernst fits for signals at the indicated g‐positions.

We recorded continuous wave EPR spectra from an oxidized CooS‐V_*Ch*_ sample poised at +62 mV and a DT‐reduced (−478 mV, all potentials vs. normal‐hydrogen electrode) CooS‐V_*Ch*_ sample (both at 10 and 80 K), as well as from a reduced and subsequently hydroxylamine‐treated CooS‐V_*Ch*_ sample (10 K) (Figures 3A and S3). All EPR spectra recorded reveal resonances at a magnetic field between 300 and 400 mT. We detected no signals at g>2.3 at *T*=10 K and high microwave power, excluding spin states S>1/2 of the detectable species. Signals of oxidized CooS‐V_*Ch*_ connected with the rhombic g‐tensor (g_*x*_=1.80, g_*y*_=1.90 and g_*z*_=1.98), are similar to that of the hybrid‐cluster of HCPs which, unlike the [4Fe‐4S]‐ and [2Fe‐2S]‐cluster, has a paramagnetic oxidized state, termed the “+5” state.^[19]^ In order to get further insight into the redox properties of the enzyme, we performed an EPR‐monitored redox titration (Figure S3‐B and S3‐C). Three sets of signals showed clear redox potential dependence and could be fitted to a Nernst curve for a one‐electron transition (Figure 3B).

We found for the species with g=[2.06 1.93 1.89] an *E*_M_=−400 mV. Although the g‐values and the relaxation (signal at 80 K still visible, not shown) are typical also for [2Fe‐2S]‐clusters, the redox potential appears to be unusually low. In other CODHs very similar g‐values, a comparable *E*_M_ and relaxation properties were found for the so‐called “B”‐cluster, a [4Fe‐4S]‐cluster.[^40^, ^41^] Therefore, we assign this signal to the [4Fe‐4S]‐cluster of CooS‐V_*Ch*_. The species with g=[2.12 1.96 1.78] is assigned to the hybrid‐cluster in accordance to the references.[^17^, ^19^] The third set of signals g=[1.98 1.90 1.80] appeared at higher E_*M*_, which we assign again to the hybrid‐cluster due to its fast relaxation at higher *T* (signal disappearance at *T*>20 K). Evaluation of the redox titration data gave midpoint potentials, which we assign to the putative redox transitions of the hybrid‐cluster as following: the “+3”/“+4” transition yielded −390 mV, the “+4”/“+5” transition −170 mV, and for the “+5”/“+6” transition we arrived at 80 mV. While the signals are very similar to those reported for HCPs, the redox potentials of the CooS‐V_*Ch*_ hybrid‐cluster are shifted to significantly more negative values (Table 1). EPR signals related to the [2Fe‐2S]‐cluster were not found over the investigated temperature and redox potential range. Additional resonances appeared upon addition of hydroxylamine to reduced CooS‐V_*Ch*_ (Figure 3A).

**Table 1:** g‐values and redox potentials for CooS‐V_*Ch*_ compared to HCPs.

Species	g‐values	*E*_M_ [mV]	Reference
Hybrid‐cluster (oxidized)	[1.98 1.90 1.80]^[a]^	−170 (“+4”/“+5”) +80 (“+5”/“+6”)	this study
Hybrid‐cluster	[15.8 13.8 6.5 5.5 2.00 1.93 1.85]^[a]^	+85 +365	[17]
Hybrid‐cluster (reduced)	[2.12 1.96 1.78]^[b]^	−390 (“+3”/“+4”)	this study
Hybrid‐cluster (reduced)	[2.09, 1.83, 1.41]^[b]^	−50	[17]
[4Fe‐4S]‐cluster	[2.06 1.93 1.89]	−400	this study

[a] (+5) state. [b] (+3) state.

### Overall Structure of CooS‐V_Ch_

**Figure 4:**
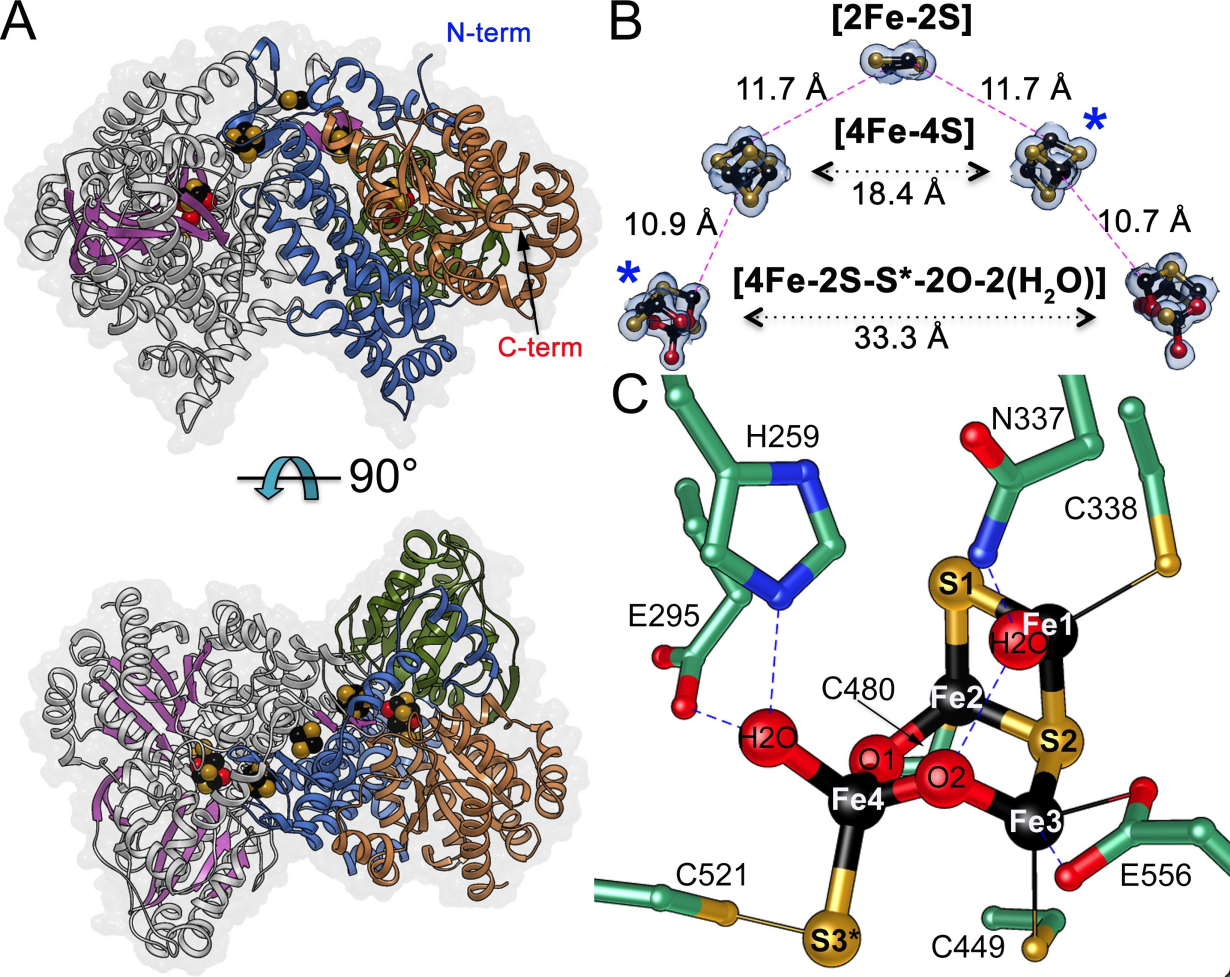
Structure of oxidized CooS‐V_*Ch*_. A) Overall structure of CooS‐V_*Ch*_ with an outline of the surface. One subunit is shown with gray helices and violet strands, the other is colored by domains (N‐terminal in blue, middle in green and C‐terminal in brown). Metal clusters are shown as spheres. B) Fe/S/O‐clusters arrangement in CooS‐V_*Ch*_. The five clusters of the dimer are shown with 1.2 rmsd of a sigma‐A weighted 2 *F*_obs_−*F*_calc_ electron density map as surface. Closest distances between iron ions are shown. Asterisks indicate clusters belonging to the same subunit. C) Oxidized [4Fe‐2S‐S*‐2O‐2H_2_O]‐cluster and its second coordination sphere. Atoms are colored: C in green, N in blue, O in red, Fe in black, S in gold. Only H‐bond lengths are indicated as dashed lines. The detailed cluster geometry is shown in Table S2 and Figure S6.

CooS‐V_*Ch*_ was crystallized under anoxic conditions and diffraction data were collected at BESSY‐II.^[42]^ We solved the crystal structure of oxidized CooS‐V_*Ch*_ using Patterson search techniques employing a truncated and modified homologous search model derived from CODH‐II_*Ch*_ (PDB‐ID: 3B53^[5]^). Two molecules were identified in the asymmetric unit and the structure was rebuilt and refined using reflections extending to 1.35 Å resolution (Table S1). Each monomer of CooS‐V_*Ch*_ has three domains, an N‐terminal (1–235), a middle (236–414) and a C‐terminal (415–629) domain (Figures 4A and S4). The N‐terminal domain is predominantly alpha‐helical and harbors one [2Fe‐2S]‐ and one [4Fe‐4S]‐cluster, whereas the middle and C‐terminal domains have a Rossmann‐fold and coordinate an Fe/S/O‐cluster.

A search for proteins of similar fold in the protein data bank using the DALI server^[43]^ revealed that CooS‐V_*Ch*_ is most similar to Ni,Fe‐CODHs, particularly to monofunctional CODHs like CODH‐II_*Ch*_ with rmsd values for Cα‐atoms below 2 Å (match of 611 of total 629 residues), but also resembles HCPs with rmsd values for Cα‐atoms of 3 Å (match of 543–553 of overall 629 residues) (Table S2).

### Iron–Sulfur Clusters in CooS‐V_Ch_

An all‐Cys bound [2Fe‐2S]‐cluster bridges the two subunits stabilizing the homodimer of CooS‐V_*Ch*_. In addition, cubane‐type [4Fe‐4S]‐clusters coordinated by the Cys residues of a C47X_2_C50X_4_C55X_12_C68 motif are bound in each subunit (Figures S4‐E and S5). The five Fe/S‐clusters of the dimer form a V‐shaped electron transfer chain with shortest edge‐to‐edge distances of ≈11–12 Å (Figure 4B).

Arrangement and type of the Fe/S‐clusters in CooS‐V_*Ch*_ are similar to CODHs and HCPs. At the same position as the bridging [2Fe‐2S]‐cluster of CooS‐V_*Ch*_, a bridging [4Fe‐4S]‐ or [2Fe‐2S]‐cluster is coordinated in the dimer interface of other CODHs, termed cluster D.[^10^, ^44^] The similarity is even more striking for the [4Fe‐4S]‐cluster, which is also found in CODHs (cluster B) and in HCP; here the Cys‐binding motif is the same as in Ni,Fe‐CODHs and differs in only one Cys position from that of HCPs (Figure S4‐E).

### Structure of the Active Site Fe/S/O‐Cluster

At the position where in CODHs cluster C is bound, we find well‐defined electron density for iron ions in the CooS‐V_*Ch*_ structure, but with an arrangement different from that of a heterocubane like cluster C. Analysis of anomalous scattering contributions of the metal cluster at wavelengths of 1.74 and 1.90 Å indicate the presence of iron and sulfur, forming a [4Fe‐2S‐S*‐2O‐2H_2_O]‐cluster in oxidized CooS‐V_*Ch*_ (Figures 4C and S5‐B). Furthermore, signals consistent with the presence of Ni were absent in X‐ray fluorescence spectra of crystalline and solution samples (Figure S5‐D and S5‐E). Two μ_2_‐bridging ligands between Fe2–Fe4 and Fe3–Fe4 were modeled as oxygen and not sulfur, based on the lack of anomalous scattering contribution, shorter Fe−O bond lengths and less electron density at this position. However, the assignment of the light atoms as oxygen is not unambiguous and neither nitrogen nor carbon or a mixture of these atoms can be excluded. Still, oxygen‐containing ligands, derived from water appear the most parsimonious interpretation. The mixed cluster can be divided into two subsites: a [2Fe‐2S‐H_2_O] site and a [2Fe‐S‐2O‐H_2_O] site, together generating two almost perpendicular planes fused along one side (106° plane angle between Fe1‐S1‐Fe2‐S2 and Fe2‐S2‐Fe3‐Fe4). The Fe1 and Fe2 atoms of the [2Fe‐2S] subsite are coordinated by Cys338 and Cys480, respectively, with Asn337 in short hydrogen‐bonding distance to the water at the [2Fe‐2S] subsite (2.4 Å). The [2Fe‐2S] subsite is similar to that of [2Fe‐2S]‐clusters with a Fe1‐Fe2 distance of ≈2.72 Å (Figure S6). The coordination of the iron atoms in the [2Fe‐S‐2O‐H_2_O] subsite is more diverse. Fe4 is coordinated by three oxygens and one sulfur: the bridging oxo/hydroxo ligands, O1 (1.64 Å) and O2 (1.98 Å), a hydroxo/water ligand (2.07 Å) and S3* (2.42 Å) that is part of a persulfide formed with the thiolate Sγ of Cys521. The water ligand is within H‐bonding distance to Glu295 and His259. Fe3 is coordinated by two oxygen and two sulfur ligands, O2 in the μ_2_‐oxo‐bridge (1.86 Å), Oϵ1 from Glu295 (2.23 Å), the thiolate of Cys449 (2.36 Å) and S2 (2.32 Å). All iron atoms in the oxidized cluster are tetrahedrally coordinated (Figure S6). With 3.69 Å for Fe2‐Fe3, 3.26 Å for Fe3‐Fe4 and 3.22 Å for Fe2‐Fe4, the Fe−Fe distances are longer than the 2.7–2.8 Å typically found in iron‐sulfur clusters. In contrast to the [4Fe‐4S]‐ and the [2Fe‐2S]‐cluster, in which all atoms could be modeled with full occupancy, the occupancies of individual atoms of the [4Fe‐3S‐2O]‐cluster were adapted to 50–70 % to refine the mixed cluster with B‐factors matching those of the surrounding ligands.

### Cluster Rearrangements upon Reduction and Turnover

Upon adding Na‐DT, oxidized crystals instantly change their color from red‐brown to pale‐orange. The crystal structure of DT‐treated CooS‐V_*Ch*_ was determined at 1.22 Å resolution (Table S1). Neither the [2Fe‐2S]‐cluster, nor the [4Fe‐4S]‐cluster underwent obvious structural changes following dithionite incubation (Figure S6). In contrast, the Fe/S/O mixed cluster in the active site rearranged considerably.

**Figure 5:**
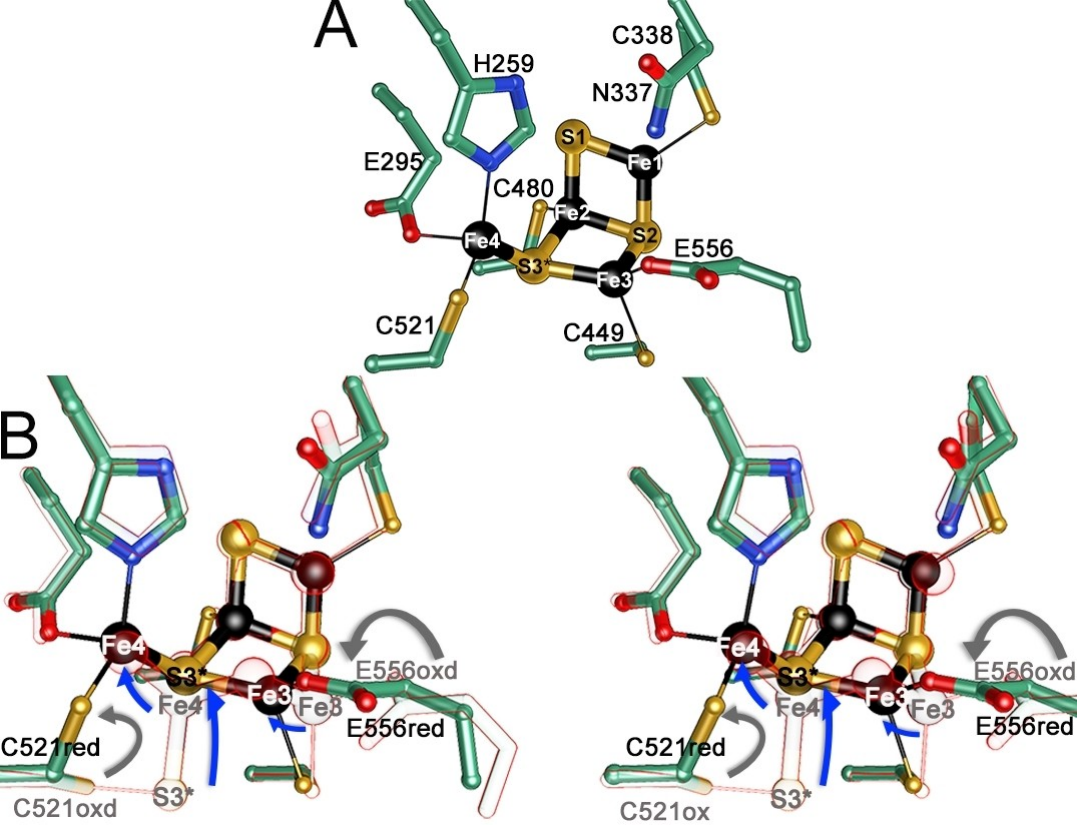
Reduced vs. oxidized active site cluster. A) Reduced [4Fe‐3S]‐cluster structure. Same color‐code and orientation as in Figure 4C are used. B) Stereoview of the superposed oxidized and reduced structures. The oxidized structure is shown with transparent balls and sticks (red‐outline). Arrows indicate movements of atoms within the cluster (blue) and of coordinating residues (gray).

Again, anomalous scattering contributions of iron and sulfur unambiguously define the composition and geometry of the reduced cluster (Figure 5A and Figure S4‐C). While the [2Fe‐2S] subsite with Fe2 and Fe3 coordinated by Cys338 and Cys480 hardly changes upon reduction, the two μ_2_‐oxo atoms and the water molecule disappeared, resulting in a [4Fe‐3S]‐cluster (Figure 5A). In the reduced state, Fe3 moves into the cluster (shift by 1.1 Å) switching the Glu556 coordination to monodentate (Figure 5B), resulting in a widening of the S2‐Fe3‐Oϵ1(E556) angle by 18° (Table S3). The S3 atom, before part of a cysteine persulfide, is now embedded in the cluster after a shift of 3.1 Å, becoming a μ_3_‐ligand for Fe2, Fe3 and Fe4. The movements of Fe3 and S3 form a new [2Fe‐2S] subsite, such that now two [2Fe‐2S] subsites are fused at an angle of 128° between planes formed by Fe1‐S1‐Fe2‐S2 and Fe2‐S2‐Fe3‐S3. Fe4, coordinated in the oxidized state by two oxo‐bridges, a sulfur and a water molecule, now relocates to an *exo* position by a 2.1 Å movement and is coordinated by His259, Glu295 and S3 and Sγ‐Cys521, for which Cys521 undergoes a 120° rotation to complete the tetrahedral coordination of Fe4. Fe1 loses its water ligand upon reduction and adopts a trigonal‐planar coordination with open coordination sites in the reduced state. This redox‐induced reorganization of the cluster is comparable to those of *D. vulgaris* HCP,^[29]^ where similar Fe−Fe distances were observed.

**Figure 6:**
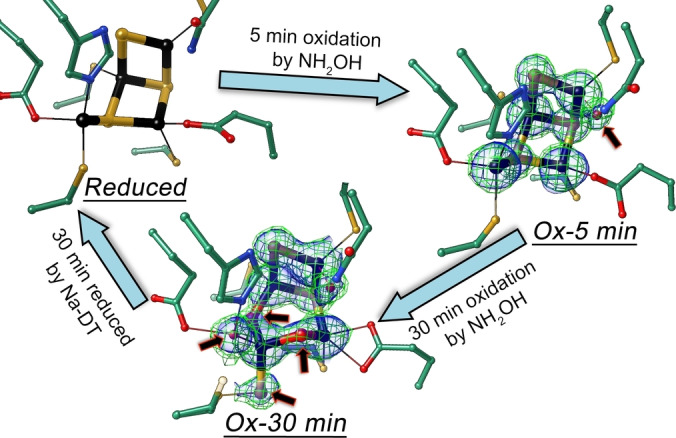
Time‐dependent structural changes of CooS‐V_*Ch*_ upon hydroxylamine incubation. Starting from the reduced structure (Reduced), oxidation of the hybrid‐cluster was observed after 5 min (Ox‐5 min) and 30 min (Ox‐30 min) of soaking the reduced crystals with 200 mM NH_2_OH. Complete redox cycling back to the reduced state was achieved by adding Na‐DT to a 30 min NH_2_OH‐incubated crystal. Sigma‐A weighted electron densities of 2 *F*_obs_−*F*_calc_ as green mesh at 1.2 rmsd and *F*_obs_−*F*_calc_ omit as blue surface at 4.0 rmsd were shown. Atoms have same color‐code as in Figure 4.

When the DT‐reduced CooS‐V_*Ch*_ crystals were incubated with the slow substrate hydroxylamine, we could follow a two‐step rearrangement of the cluster. In the first step (Ox‐5 min in Figure 6), Fe1 obtains again a water ligand (arrow in Ox‐5 min), similar to the oxidized structure, while the rest of the cluster is unchanged. Longer incubation of the reduced crystals with hydroxylamine (Ox‐30 min in Figure 6) forms the two oxo‐bridges between Fe4‐Fe2 and Fe4‐Fe3 and rearranges Fe4 and S3 (arrows in Ox‐30 min), so that the cluster returns to its fully oxidized structure, as observed in the crystal treated with 10 mM ferricyanide (Figure 4C). Subsequent reductive cycling of the hydroxylamine‐oxidized crystal with Na‐DT restores the reduced state of the cluster (Reduced in Figure 6). These rearrangements indicate that I) all changes in cluster geometry are reversible, II) at least three (structural) states of the hybrid‐cluster can be distinguished and III) Fe1 with its unusual trigonal planar coordination in the reduced state is the first metal to change its coordination. Fe1 together with Fe3 and Fe4 is also the likely place for substrate activation.

Notably, while the four additional ligands of the oxidized state were modeled as O(H)_*x*_, they may partly derive from the reduced hydroxylamine and include a nitrogen‐containing ligand.

### Channels for Small Molecules

**Figure 7:**
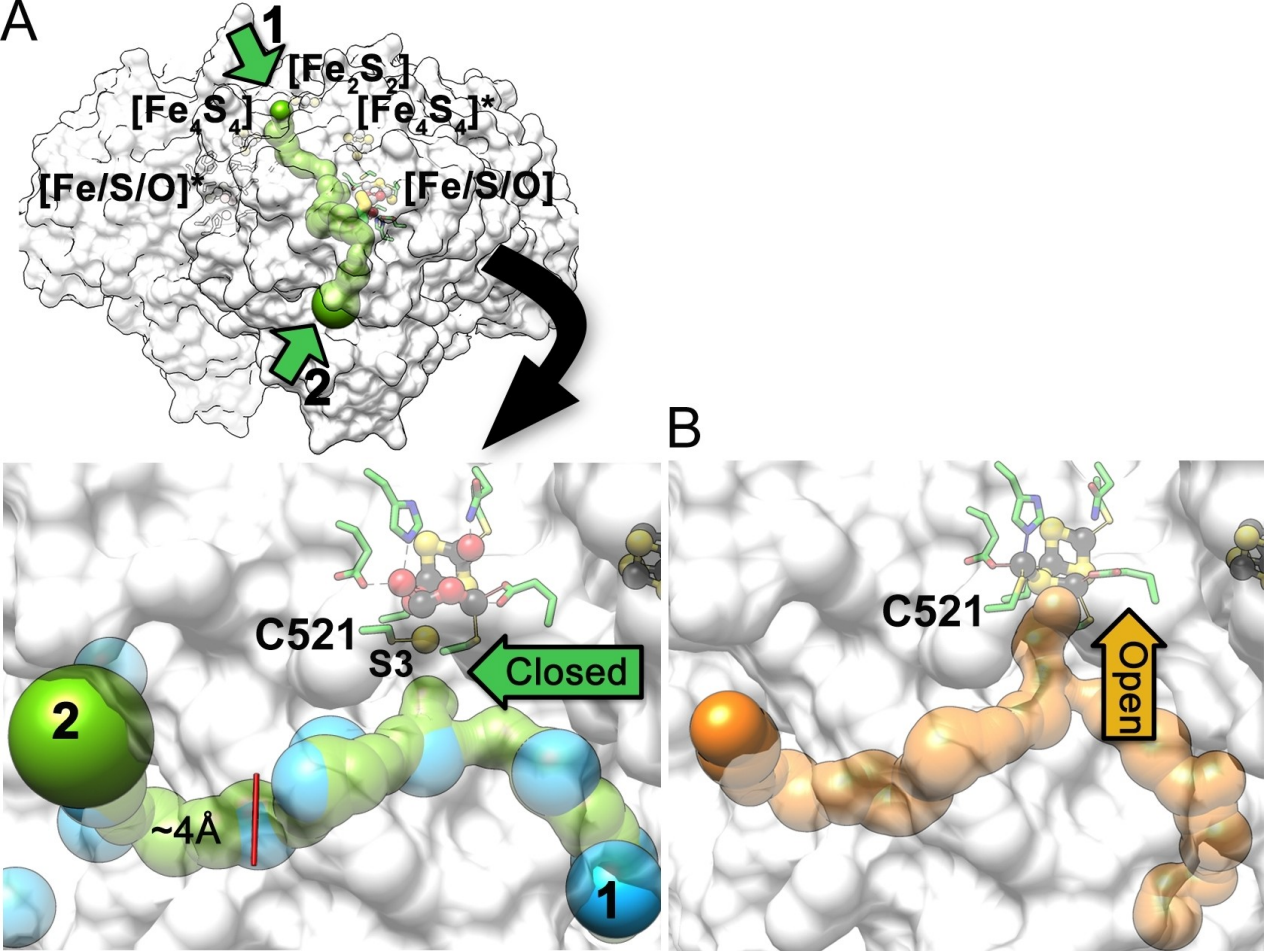
Redox‐dependent access to active site. A) Two channels (numbered 1 and 2 shown as green surfaces) converge at the oxidized hybrid‐cluster. Xe atoms (blue spheres) from the Xe‐derivatization are shown. A movement of the S3 atom with the coordinating C521 (marked) blocks the channel at the end of the cluster. B) Channels (orange surface) in the reduced structure. Same orientation as in A.

Potential gas channels around the hybrid‐cluster were calculated for the oxidized and reduced structures of CooS‐V_*Ch*_ using MOLE.^[45]^ In the oxidized state, two channels with widest diameter of approx. 4 Å converge near the hybrid‐cluster (1 and 2 in Figure 7A upper panel). However, there is no access to the hybrid‐cluster in the oxidized state, because the S3 atom, now part of a persulfide, blocks the path to the cluster (closed conformation in Figure 7A). When the hybrid‐cluster is reduced, it rearranges so that the persulfido sulfur becomes a μ_3_‐bridging ligand within the cluster (Figure 5). This switch of position for S3 and Sγ‐Cys521 opens the channel, providing access to the hybrid‐cluster cavity (open conformation in Figure 7B). Additionally, we pressurized CooS‐V_*Ch*_ crystals with Xe to probe hydrophobic channels. Using the anomalous scattering contribution of Xe atoms at a wavelength of 1.90 Å, 64 Xe atoms were located in the four molecules of the asymmetric unit in which each molecule has approx. 15 Xe atoms bound (Figure S7‐A). When the four molecules with their bound Xe atoms are superimposed, common positions of Xe atoms in the interior of the structure fit well to the calculated channel (Figure 7A). This redox‐dependent channel opening and closing of CooS‐V_*Ch*_ suggests that substrate(s) may only bind to the reduced state of the hybrid‐cluster and that therefore CooS‐V_*Ch*_ may act as a reductase.

### CooS‐V_Ch_ and CODHs

**Figure 8:**
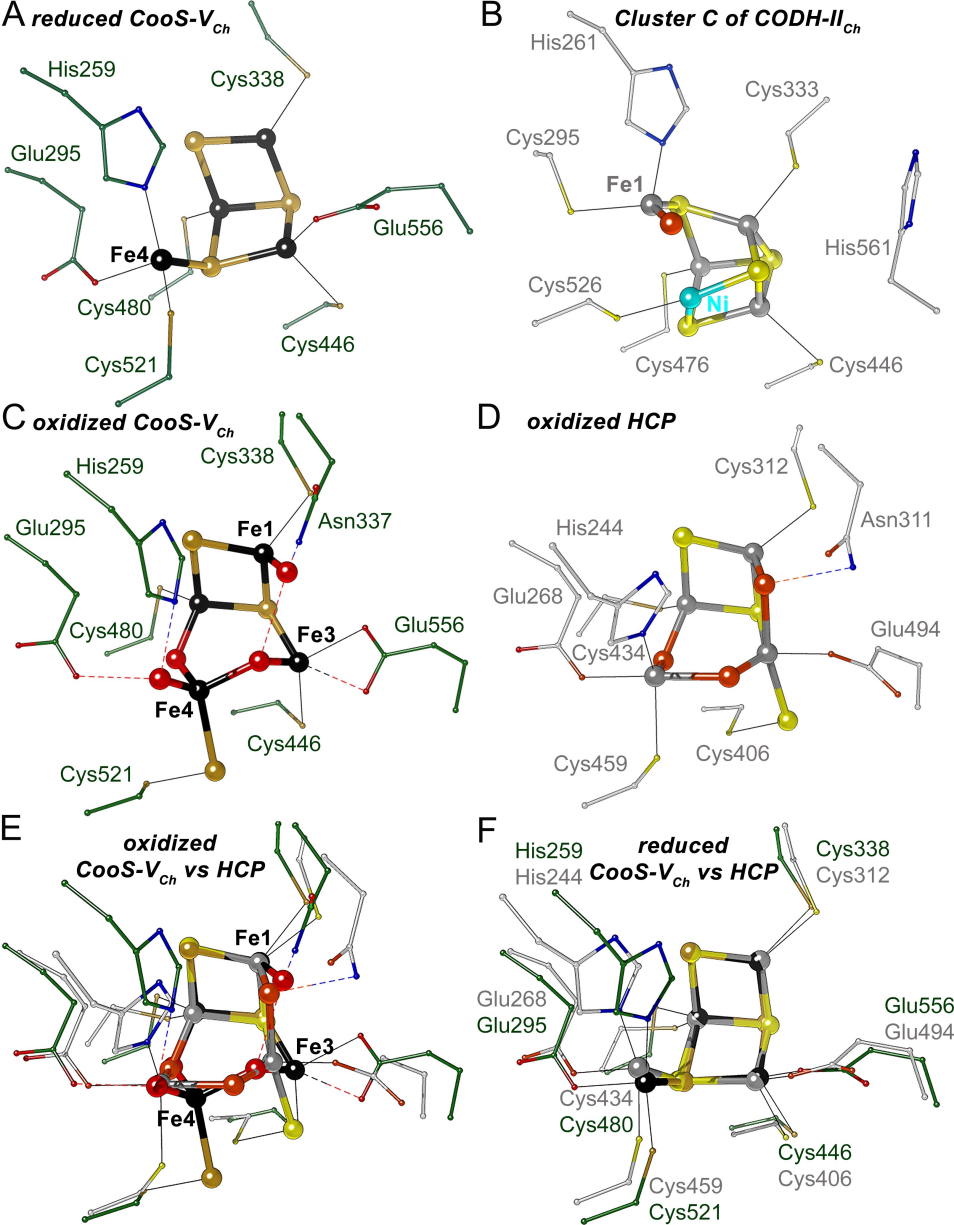
Active site structure comparison. A) Comparison of the reduced hybrid‐cluster of CooS‐V_*Ch*_ to (B) the [Ni4Fe‐4S‐H_2_O]‐cluster C of CODH‐II_*Ch*_. C) The oxidized hybrid‐clusters of CooS‐V_*Ch*_, D) HCP and E) superposition of them. F) Superposition of the reduced hybrid‐clusters of CooS‐V_*Ch*_ and HCP. Residues in E are positioned as in D. Cluster C and the hybrid‐clusters are shown as ball‐and‐stick. Color code for CooS‐V_*Ch*_ is identical as in Figure 3. Atoms of HCP and CODH‐II_*Ch*_ are colored: C in light gray, S in yellow, N in blue, O in orange‐red, Ni in cyan, Fe in dark gray. PDB‐IDs: 3B53 for CODH‐II_*Ch*_,^[5]^ 1OA1 for reduced HCP^[30]^ and 1W9M for oxidized HCP^[26]^ were used. For stereo‐view see Figure S9.

CooS‐V_*Ch*_ and homologous proteins with the same sequence motifs as CooS‐V_*Ch*_ are annotated in sequence databanks as Ni,Fe‐CODHs. This is not surprising, as CooS‐V_*Ch*_ is most similar to Ni,Fe‐CODHs—both by sequence and structure similarity. When aligning the amino acid sequences and structures of CooS‐V_*Ch*_ and CODH‐II_*Ch*_, it is striking that of the six residues coordinating cluster C in CODH‐II_*Ch*_ only Cys295 is not conserved and is a glutamate in CooS‐V‐type sequences (Figure 8A, B). In CODHs, Cys295 (CODH‐II_*Ch*_ numbering) coordinates Fe1, the Fe^II^ ion close to Ni in *exo* position to the [Ni3Fe]‐heterocubanoide of cluster C.[^4^, ^7^] The C295A and C295E variants of CODH‐II_*Ch*_, when produced in *Escherichia coli*, resulted in a Ni‐deficient enzyme unable to oxidize CO, but with a slightly increased NH_2_OH reductase activity.^[46]^ The exchange of Cys295 for Glu is probably a prerequisite to form the hybrid‐cluster rather than cluster C and it appears to be a synapomorphic character state of the subclade to which CooS‐V_*Ch*_ belongs (Figure 2B). The exchange of Cys295 to Glu correlated with the exchange of Gly41 to Cys41 in the CooS‐V subclade (Figure S8). Cys41 coordinates the subunit‐bridging [2Fe‐2S]‐cluster in CooS‐V_*Ch*_ (Figure S5‐A). Although this may indicate co‐evolution of both amino acid exchanges, the Gly>Cys exchange evolved independently and in parallel in the more distantly related CODHs, like the CODH of *D. vulgaris* belonging to cluster I, where it also resulted in the formation of a [2Fe‐2S]‐cluster.^[10]^ Interestingly, the presence of a [2Fe‐2S]‐cluster has been implicated in increasing the dioxygen‐tolerance of the *D. vulgaris* CODH compared to the [4Fe‐4S]‐cluster.^[11]^ Thus, the Gly>Cys exchange may have been fixed in CODH lineages, once a resistance to oxidative or nitrosative stress became important.

### CooS‐V_Ch_ and HCPs

While the hybrid‐clusters found in CooS‐V_*Ch*_ and HCP are similar, they show some remarkable structural and functional differences. While the structure of the reduced [4Fe‐3S]‐cluster of both proteins is very similar, the oxidized states differ at several places (Figure 8C–F). The most obvious differences in the oxidized state are the ligands of Fe4 and Fe3. While Fe4 has a trigonal‐bipyramidal coordination in HCP with three protein ligands (Cys459, His244 and Glu268), it has a tetrahedral coordination in CooS‐V_*Ch*_ and no protein‐ligand—it is only indirectly coordinated by the protein matrix via the persulfido‐ligand at Cys521 (Figure 8C). The terminal water ligand of Fe4 in CooS‐V_*Ch*_ is not found in HCPs. A persulfido‐ligand is also present in oxidized HCP, but there it is bound to Fe3, which is fivefold‐coordinated (2×S; 3×O) with a distorted trigonal‐bipyramidal arrangement, while in CooS‐V_*Ch*_ Fe3 has a tetrahedral coordination (2×S; 2×O) (Figure 8C–E). The water ligand at Fe1, which is visible already after 5‐min reaction with hydroxylamine and remains at its place for the complete oxidation, is a terminal ligand in CooS‐V_*Ch*_ and a bridging ligand in HCP. One consequence of the differences in the oxidized states of the hybrid‐clusters is the oxidation‐state dependent opening and closing of the active site cavity in CooS‐V_*Ch*_, which is determined by the position of the persulfide (Figure 7). The underlying reasons for these differences are unclear, but may be linked to the conformation of Glu556 that changes upon oxidation in CooS‐V_*Ch*_, while the corresponding residue in HCP (Glu494) remains unchanged upon oxidation. Another reason might be that we may have generated the “+6” state using ferricyanide, while the oxidized HCP structures were reported for the “+5” state.[^47^, ^48^, ^49^] However, we observed the same structure of the metal cluster after incubating the reduced crystal for 30 min with the oxidant hydroxylamine (Figure 6).

The observed structural differences are probably also linked to the more negative reduction potential of hybrid‐cluster in CooS‐V_*Ch*_. Compared to the hybrid‐cluster of the HCP from *E. coli* all three midpoint potentials are about 300 mV more negative in CooS‐V_*Ch*_ (Table 1). This would support catalytic reactions to occur at much more negative redox potentials, suggesting different physiological functions for HCPs compared to CooS‐V type enzymes.

## Conclusion

Geobiochemical and phylogenetic analysis agree with the antiquity of the reductive acetyl‐CoA pathway and with the catalytic role of Ni/Fe/S‐clusters in general and in Ni,Fe‐CODHs in particular.[^13^, ^14^] Therefore, the common ancestor of Ni,Fe‐CODHs and HCPs might have CODH activity and likely contained a Ni/Fe/S‐cluster similar to cluster C. In this scenario, proteins containing a hybrid‐cluster evolved at least two times from a CODH scaffold: at an early time, resulting in the large group of HCPs found in bacteria, archaea and eukarya, and at a later time, evolving the hybrid‐cluster found in CooS‐type enzymes. The amazing similarity between CooS‐V_*Ch*_ and CooS‐type CODHs shows that little changes suffice to generate a functional, reversibly restructuring hybrid‐cluster with low redox potential within a CODH protein matrix.

## Conflict of interest

The authors declare no conflict of interest.

1

## Supporting information

As a service to our authors and readers, this journal provides supporting information supplied by the authors. Such materials are peer reviewed and may be re‐organized for online delivery, but are not copy‐edited or typeset. Technical support issues arising from supporting information (other than missing files) should be addressed to the authors.

Supporting Information

## Data Availability

All structures and structure factors are available from https://www.rcsb.org under the identifiers: 7B7Q, 7B7T, 7B95, 7B97, 7B9A.
