# Supplementary material for: A Morphing [4Fe‐3S‐nO]‐Cluster within a Carbon Monoxide Dehydrogenase Scaffold
Source: Angew Chem Int Ed Engl. 2022 Mar 4;61(18):e202117000. doi: 10.1002/anie.202117000 (PMC9311411; doi:10.1002/anie.202117000)
Supplement: Supplementary file 1 — Supporting Information [file ANIE-61-0-s001.pdf]

Angewandte Chemie
Eine Zeitschrift der Gesellschaft Deutscher Chemiker

Supporting Information

A Morphing [4Fe-3S-nO]-Cluster within a Carbon Monoxide Dehydrogenase Scaffold

J.-H. Jeoung, J. Fesseler, L. Domnik, F. Klemke, M. Sinnreich, C. Teutloff, H. Dobbek**

Table of Contents

Experimental Procedures 3

Supplementary Tables 6
Table S1. Crystallographic Data Collection and Refinement Statistics 6
Table S2. Structures similar to CooS-V_{Ch} detected using DALI 8
Table S3. Angles in the Active Site Clusters of CooS-V_{Ch} 9

Supplementary Figures 10
Figure S1. Phylogeny of CooS-type CODHs 10
Figure S2. Biochemical Characterization of CooS-V_{Ch} 11
Figure S3. EPR Spectroscopy 12
Figure S4. Topology of CooS-V_{Ch} 13
Figure S5. Electron Density Representation 15
Figure S7. Analysis of Channels 16
Figure S8. Mirror Tree Cladogram for the History of Cys41 and Glu295 17
Figure S9. Stereo View of Figure 7 18

References 19

Author Contributions 20

Experimental Procedures

Materials. All chemicals purchased were at least of analytical grade. N_2 and N_2/H_2 (95%/5%) were purchased from Air Liquide. Enzymes (DNA Polymerase, DNA ligase, and restriction enzymes) used in molecular biology were obtained from either Fermentas or New England Biolabs. All anoxic solutions were prepared in bottles with butyl rubber septum by successive cycles (at least four cycles) of evacuating and flushing with N_2 gas at a vacuum-gas line. Purification, crystallization and all other biochemical experiments were performed under anoxic conditions under an atmosphere of 95% N_2/5% H_2 inside a glove box (model B; COY Laboratory Products, Inc.).

Bioinformatic Methods. InterPro entry IPR004137 contained originally 9634 amino acid sequences[1] from which unclassified sequences and sequences derived from metagenomes were deleted, the sequence of CooS-V_{Ch} was added, leaving 7466 entries. From sequence pairs sharing Kimura distances smaller than 0.3, one entry was removed, yielding a dataset with 1270 sequences. Sequences were iteratively aligned and a distance matrix based on Kimura distances was used to construct the sequence similarity network[2] in which all sequences with a Kimura distance below 1.0 are connected. Cytoscape 3.8[3] was used for visualization. Sequences for clusters I to IV were combined, entries containing less than 550 amino acids were removed and the remaining 386 sequences were iteratively aligned using mafft Version 7.453 using the L-INS-i strategy.[4] ProtTest 3[5] was used to estimate the substitution model with highest corrected Akaike information criterion. PHYML 3.3[6] with the LG model[7] and gamma-distributed among site rate variations in four categories together with empirical state frequencies was used to infer a maximum-likelihood tree with a Bayesian-like approximate likelihood ratio test to estimate individual branch supports[8]. The minimal ancestor deviation method[9] was used to root the tree. Codeml (PAML 4.9d)[10] was used for a marginal reconstruction of the posterior probability distributions of ancestral states using the empirical Bayes maximum-likelihood approach. We used Mesquite version 3.6.1[11] to trace and display character evolution calculating parsimony ancestral states.

FigTree 1.4.4[12] was used to display phylogenies. Channels were calculated using MOLEonline (version: Mole 2.5).[13] Figures showing structures were prepared using UCSF Chimera version 1.12.[14]

Cloning and Expression. The gene CHY_RS00160[15] annotated as coding for CODH-V in *Carboxydothermus hydrogenoformans* (CooS-V_{Ch}) was amplified by PCR with a pair of primers including *Bsa*I restriction sites using Phusion DNA polymerase: 5'-GGAGATGGCGCCGCTACAAAAACGTCCATTCA-3' (forward) and 5'-GGAGATGTATCATTAAATACCCAGTTTGGCGC-3' (reverse). The resulting ~1.9 kbp DNA fragment was digested with *Bsa*I and ligated into a *Bsa*I-digested pASK-IBA 17-plus vector (IBA, Göttingen Germany). After transformation of the ligation products into *Escherichia coli* DH5α, a plasmid with the insert was analyzed by restriction digest and sequencing (Eurofins Genomics Germany GmbH), termed pPK-S5-strep.

For expression of the gene, pPK-S5-strep was transformed into *E. coli* strain BL21 (DE3). Cultures were grown at 37 °C in modified TB media[16] supplemented with 50 µg/ml kanamycin, 1% (w/v) glucose, 0.2 mM $FeSO_4$ and 0.2 mM Na_2S in 5-L glass fermenters under stirring and access of air. After the OD_{600} reached a value of 0.4, the bottles were sealed with butyl rubber septa to restrict oxygen contact. The culture was further stirred until an OD_{600} of 0.7-0.8 was reached. Gene expression was stimulated by addition of 0.2 mg/l anhydrotetracycline and continued for 16 h at 30 °C. Cell pellets were stored at -80 °C until further use.

Purification. Frozen cells were resuspended in buffer S (50 mM Tris-HCl pH 8.0 and 300 mM NaCl) with 2 mg/L avidin and stirred for 30 min. N-octyl-β-D-maltoside was subsequently added until a final concentration of 0.5% (w/v) was reached. Cell lysis was achieved by sonication in an ice-cooled rosette cell. A clear, soluble fraction was obtained by ultracentrifugation at 95,800 *g* at 12 °C for 1 h. The supernatant was loaded onto a Strep-Tactin® Superflow® high capacity column (IBA, Göttingen Germany) equilibrated with buffer S, followed by extensive washing with ten column volumes of the same buffer. Protein was eluted with buffer E (buffer S + 2.5 mM desthiobiotin) in a volume of 20 mL and subsequently concentrated. Protein buffer was exchanged versus buffer G (20 mM Tris-HCl pH 8.0) on a PD-10 desalting column (GE Healthcare).

The N-terminal Strep-Tag was removed by treatment with strep-tagged TEV (tobacco etch virus) protease in 100-fold excess with 5 mM β-mercaptoethanol overnight at 25 °C. Strep-tag free CooS-V_{Ch} was isolated by collecting the flow-through fraction after loading on to the Strep-Tactin column. A PD-10 column was used to exchange buffer with 20 mM Tris-HCl pH 8.0. The protein was frozen in glass vials equipped with butyl rubber septum in liquid N_2 and stored at -80 °C

Fe and Protein Quantification. The non-heme iron content was determined using the published method.[17] Protein concentration was routinely measured according to Bradford[18] using the reagent from Roth (Roti®-Quant).

Spectroscopic Methods. UV-vis spectra of CooS-V_{Ch} were recorded on an Agilent 8453 photodiode array spectrophotometer. All measurements were performed anaerobically at room temperature using quartz cuvettes with a path length of 1 cm. Potassium ferricyanide was used to oxidize the protein and was subsequently removed by buffer exchange on PD-10 column. To obtain a reduced spectrum, the oxidized protein (18 µM in 50 mM Tris-HCl pH 8.0 and 100 mM NaCl) was titrated by Na-dithionite (DT) in 1 µM steps.

Electron Paramagnetic Resonance (EPR) Spectroscopy. Samples for EPR spectroscopy were prepared in the anoxic glove box. For the redox titration the oxidized/as-isolated protein (200 µM, Tris-HCl pH 8) was transferred into a dedicated redox cell equipped with a Pt redox electrode and continuous solution mixing. The protein solution was stepwise reduced by addition of Na-DT (1-10 µM).

Redox potential stabilization was achieved by including a redox mediator mixture in the solution. When the potential had stabilized, about 100 µl of protein solution was transferred into an EPR tube for EPR measurements. Continuous wave EPR spectra were recorded at 10 and 80 K on a home built X-band spectrometer featuring a Bruker ER 041 MR microwave source, a Bruker ER 4122-SHQ E resonator optimized for cw-measurements, a Stanford Research Systems SR810 lock-in amplifier, an AEG electromagnet, driven and controlled by a Bruker ESR 2388 power supply and a Bruker B-H 15 field controller. Temperature control was achieved using an Oxford Instruments ESR 910 cryostat attached to the resonator.

NH_2OH-Reduction and CO-Oxidation Assay. Hydroxylamine reduction activity was assayed as previously described by Wolfe *et al.*[19] monitoring the oxidation of reduced methyl viologen (MV_{red}). In 2 mL quartz cuvettes, a buffer (100 mM HEPES pH 8.0 and 100 mM NaCl) was added and incubated for 2 min at 40 °C, before a base line spectrum was recorded. Subsequently added oxidized MV (final concentration: 10 mM) was reductively titrated with dithionite solution, until a final absorption of 1.2 at 578 nm (A_{578}) was obtained. The concentration of hydroxylamine was varied from 0.5 to 100 mM. When no significant changes in absorbance were observed, the reaction was started by adding 1 µM CooS-V_{Ch} and monitored for 300 s. Initial rates were determined and specific activities calculated using the extinction coefficient of MV_{red} at 578 nm (ε_{578} = 9.7 $mM^{-1}cm^{-1}$). The steady-state kinetic constants K_m and k_{cat} were determined by fitting the experimental data with a nonlinear regression applying the Michaelis-Menten equation in GraFit 5.[20]

CO-oxidation activity was measured as described previously.[16]

NO Reductase Activity Assay. We used two different assays to monitor NO reductase activity of CooS-V_{Ch}: 1) detecting NADH oxidation by the enzyme hybrid cluster protein reductase (HCR) from *E. coli* by time-dependent absorbance measurement at 340 nm and 2) use of gas chromatography (GC) and mass spectrometry (MS) to detect NO consumption and N_2O production from the gas phase of the reaction vessel. NO gas was purged through 1 M NaOH solution and quantified by formation of a NO-myoglobin complex with an ε at 421 nm (114 mM^{-1} cm^{-1}).[21]

1) Time-dependent absorbance at 340 nm. The gene encoding HCR from *E. coli* BL21 (DE3) was cloned into a pET28 vector and HCR was aerobically expressed in *E. coli* BL21 (DE3) using an autoinduction medium.[22] HCR was purified by affinity chromatography using the N-terminal his-tag according to the manufacturer's recommendations (GE Healthcare). After the reconstitution, HCR possessed 1.9 Fe and 1.2 FAD per mole of protein.

We followed the reduction of CooS-V_{Ch} (0.2 µM) by adding HCR (2.5 µM) with 250 µM NADH as reducing agent, which was monitored by the decreasing absorption at 450 nm. The observed rate constant (k_{obs}) of reduction of CooS-V_{Ch} by HCR of 50/min was determined by fitting a single-exponential function.

To measure NO reduction activity, 40 nM CooS-V_{Ch} and 160 nM HCR in 50 mM Tris-HCl pH 8.0 were mixed in a gas-tight cuvette without any gas phase. The reaction was started by adding ~240 µM NO solution and the decrease in NADH concentration was monitored at 340 nm. NADH consumption was quantified using an ε_{340nm} of 6.22 mM^{-1} cm^{-1}.

2) Use of GC/MS. We adapted our assay[23] with a modification of the SIM mode (m/z=30 for NO and m/z=44 for N_2O) to detect NO and N_2O in the reaction vessel by MS. Two reduction methods were used: with HCR and NADH as in (1) and benzyl viologen and Na-DT as reductants. The reaction contained 40 nM CooS-V in 50 mM Tris-HCl pH 8.0 with either 150 nM HCR and 600 µM NADH or 5 mM Na-DT and 1 mM benzylviologen as reducing agent in 1 ml solution with 2 ml of gas phase. 100 µl of gas phase was injected into the GC/MS at different times during the reaction.

We also performed different control experiments: 1. Only HCR and NADH, 2. Only NADH, 3. Only benzylviologen and Na-DT without CooS-V_{Ch}.

Crystallization and Crystal Treatment. CooS-V_{Ch} crystals were obtained by hanging-drop vapor diffusion and were grown in 0.1 M Bis-Tris propane (pH 6.5), 0.15 M KSCN and 16-20% (w/v) polyethylene glycol (PEG) 3350 by mixing of equivalent volumes (2 µl) of reservoir solution with 15 mg/ml CooS-V_{Ch} in 20 mM Tris-HCl pH 8.0 at 18°C. Crystals in the as-isolated state were harvested and incubated for 10 min in mother liquor with 10 mM potassium ferricyanide to oxidize the crystal. Crystals in the reduced state were obtained after soaking the oxidized crystal in the mother liquor with addition of 15 mM Na-DT for 30 min. The DT-reduced crystals were thoroughly washed with the mother liquor without Na-DT and incubated with 200 mM NH_2OH for 5 min and 30 min. A crystal from the latter state was reduced back using 15 mM Na-DT.

Crystals for Xe-derivatization were obtained by hanging-drop vapor diffusion in a condition of 0.1 M Bis-Tris propane (pH 7.5), 0.2 M NaBr and 20% (w/v) PEG3350 by mixing of equivalent volumes (2 µl) with 15 mg/ml CooS-V_{Ch} in 20 mM Tris-HCl pH 8.0 at 18°C. Xe-derivatized crystals were prepared by incubation in a noble gas chamber (Hampton Research, USA) with Xenon gas (99.99% purity, Linde Berlin Germany) at 27 bar for 7 min. Crystals were immediately flash-cooled in liquid nitrogen, after the pressure was slowly released.

All crystals were flash-cooled in liquid N_2 with 15% (v/v) 2R,3R-butanediol as a cryo-protectant.

Data Collection, Structure Determination and Refinement. Diffraction data from crystals cooled at 100 K were collected on beamlines BL 14.1 and 14.2 (BESSY, Berlin, Germany).[24] To position Fe and S atoms in the reduced and oxidized states and Xe atoms in the derivatized crystals, datasets were collected at the high-energy side of the K-absorption edge of Fe (λ = 1.74 Å), S (λ = 1.90 Å) and close to the L_I-absorption edge of Xe (Xe-L_I-edge: 2.27 Å, measured at λ = 1.90 Å) for the calculation of an anomalous difference Fourier map.

Diffraction data were integrated and scaled using XDSAPP.[25] Initial phases were obtained from Patterson search techniques with AutoMR in Phenix[26] using the structure of CODH-II_{Ch} in the –320 mV state (PDB-ID: 3B53[16]) as a homologous search model. Several cycles of automated model building and refinement were carried out with AutoBuild in Phenix.[27] After iterative manual model building using Coot,[28] further refinements were performed using REFMAC5[24] of the CCP4 suite[16] and phenix.refine[29].

In the crystal batch used for Xe-derivatization, the length of the b-axis had doubled compared to the batch of crystals used for structure determination, resulting in four CooS-V_{Ch} molecules in the ASU. As the Br ions from the crystallization condition show weak anomalous scattering, not all anomalous scattering contributions derive from Xe atoms. 23 positions with anomalous scattering in the Xe-derivatized structure were modeled with Br ions, as the structure determined before Xe-derivatization showed Br ions at the same positions. In total, 64 Xe atoms were modeled according to their strong anomalous scattering contribution in the ASU (Figure S7-A).

Data collection and refinement statistics of the reported structures are given in Table S1. Coordinates and structure factor amplitudes have been deposited in the Protein Data Bank under accession numbers 7B7Q.pdb (oxidized), 7B7T.pdb (reduced), 7B95.pdb (NH_2OH-5 min), 7B97.pdb (NH_2OH-30 min), and 7B9A.pdb (Xe-derivatized).

Supplementary Tables

Table S1. Crystallographic data collection and refinement statistics.

	**S5-*oxd***	**S5-*red***	**S5-NH_2OH: 5 min**	**S5-NH_2OH: 30 min**	**Xe-derivative**
**Data collection**					
Wavelength (Å)	0.91841	0.91841	0.91841	0.91841	1.90
Space group	$P2_1$	$P2_1$	$P2_1$	$P2_1$	$P2_1$
Cell constant					
a, b, c (Å)	66.33, 108.98, 82.84	65.99, 108.02, 82.3	65.15, 108.07, 82.24	65.20, 108.17, 82.23	69.41, 230.05, 82.74
α, β, γ (°)	90.0, 98.8, 90.0	90.0, 98.9, 90.0	90.0, 94.8, 90.0	90.0, 95.2, 90.0	90.0, 101.5, 90.0
Nr. of molecules in asu	2	2	2	2	4
Unique reflections[a]	251 838 (25 173)	228 651 (22 516)	220 775 (21 847)	194 715 (18 875)	80 177 (8 105)[c]
Redundancy	5.1	6.8	6.7	6.9	3.8
R_{meas} (%)[a]	5.0 (123.3)	8.3 (124.9)	7.4 (117.1)	6.3 (119.2)	7.1 (14.4)
$R_{CC1/2}$ (%)[a]	99.9 (56.4)	99.9 (59.9)	99.9 (66.7)	100 (68.5)	95.2 (92.9)
Resolution (Å)[a]	50.0-1.35 (1.40-1.35)	50.0-1.38 (1.43-1.38)	50.0-1.40 (1.45-1.4)	50.0-1.45 (1.05-1.45)	20.00-2.50 (2.59-2.5)
Completeness (%)[a]	99.06 (99.34)	98.78 (97.41)	99.33 (98.83)	97.19 (94.66)	91.6 (92.9)
I/sigma *I*[a]	15.1 (1.1)	12.8 (1.5)	13.4 (1.5)	15.6 (1.4)	14.7 (7.4)
**Refinements**					
Resolution (Å)	45.36-1.35	47.38-1.38	45.11-1.40	48.75-1.45	19.99-2.50
Reflections used in refinement	251 822	228 647	220 766	194 694	80 142
Model R_{work}/R_{free} (%)[b]	13.40/15.95	13.12/15.92	13.33/16.62	13.46/17.12	23.24/23.09
Ramachandran-statistics (%)					
favored/allowed/disfavored	97.20/2.64/0.16	97.20/2.56/0.24	97.21/2.55/0.24	97.05/2.71/0.24	94.93/4.91/0.16
Wilson B	17.51	14.16	14.87	17.77	25.28
B-factors					
Protein/water	22.87/37.43	18.76/34.27	20.37/34.14	23.98/37.25	25.95
[2Fe-2S]/[4Fe-4S]	16.65/11.43	12.80/11.57	12.59/12.31	15.29/14.17	12.32/16.20
[Hybrid-cluster]	13.35	15.26	14.57	23.46	34.05
rmsd of ideal geometry					
Bond (Å)/Angle (°)	0.0111/1.240	0.0137/1.531	0.0128/1.457	0.010/1.124	0.003/0.585

Table S1 continued.

	S5-oxd/Fe-peak	S5-oxd/S-peak	S5-red/Fe-peak	S5-red/S-peak
**Data collection**				
Wavelength (Å)	1.71	1.90	1.71	1.90
Space group	$P2_1$	$P2_1$	$P2_1$	$P2_1$
Cell constant				
a, b, c (Å)	66.33, 108.84, 82.74	65.23, 108.69, 82.63	66.07, 108.10, 82.27	66.06, 108.07, 82.25
α, β, γ (°)	90, 98.83, 90	90, 98.84, 90	90, 99.08, 90	90, 99.09, 90
Total/unique reflections	1 353 515/110 020[c]	958 929/77 096[c]	1 231 248/100 660[c]	939 780/77 042[c]
R_{meas} (%)[a]	6.1 (149.1)	5.0 (31.0)	5.2 (18.7)	3.9 (9.2)
$R_{CC1/2}$ (%)[a]	100 (68.5)	100 (96.7)	99.9 (98.9)	100 (99.7)
Resolution (Å)[a]	49.42-1.77 (1.84-1.77)	47.64-2.00 (2.07-2.00)	47.37-1.82 (1.89-1.82)	49.24-1.99 (2.06-1.99)
Completeness (%)[a]	98.2 (98.4)	98.81 (98.77)	98.64 (97.11)	98.65 (96.97)
I/sigma *I*[a]	25.2 (1.1)	33.0 (5.9)	35.0 (10.3)	44.6 (19.0)

asu, asymmetric unit; oxd, oxidized; red, reduced

[a], Values in parentheses are given for the highest-resolution shell.

[b], R_{free} factor was calculated from 5% of randomly selected data before refinement was carried out.

[c], Friedel pairs are treated as different reflection.

asu, asymmetric unit

Table S2. Structures similar to CooS-V_{Ch}, searched using DALI.[29] Only representative structures with Z-score higher than 20 with less than 3 Å of rmsd are selected in the table. ACS, acetyl-CoA synthase; ACDS, acetyl-CoA decarboxylase/synthase.

PDB-ID	Z-score	Rmsd (Å)	Identity (%)	Description
6ELQ	50.5	1.6	34	CODH-IV from *C. hydrogenoformans*
3B53	49.9	1.5	32	CODH-II from *C. hydrogenoformans*
1JQK	48.6	1.5	31	CODH from *Rhodospirillum rubrum*
1OAO	48.4	1.5	28	bifunctional CODH/ACS from *Moorella thermoacetica*
1UPX	28.0	3.0	20	HCP from *Desulfovibrio desulfuricans*
1OA1	27.8	2.7	19	HCP from *D. vulgaris*
3CF4	23.1	2.9	28	CODH of ACDS from *Methanosarcina barkeri* *

* While the CODHs from *C. hydrogenoformans*, *R. rubrum* and *M. thermoacetica* are CooS-type CODHs, the CODH from *M. barkeri* is a Cdh-type CODH.

Table S3. Angles in the active site clusters of CooS-V$_{Ch}$. Average values from two molecules in asymmetric unit are used. Atomic numbers are same as in Figure S6-C and -D.

Atoms	Oxidized Angle (°)	Reduced Angle (°)
S^1-Fe^1-S^2	107	109
S^1-Fe^1-$S_\gamma(C^{338})$	117	127
S^1-Fe^1-H_2O	108	
S^2-Fe^1-$S_\gamma(C^{338})$	114	123
H_2O-Fe^1-$S_\gamma(C^{338})$	102	-
S^2-Fe^1-H_2O	108	-
S^1-Fe^2-S^2	103	105
S^1-Fe^2-O^1	121	-
S^1-Fe^2-Fe^4	111	-
S^1-Fe^2-$S_\gamma(C^{480})$	101	99
S^2-Fe^2-$S_\gamma(C^{480})$	116	116
O^1-Fe^2-$S_\gamma(C^{480})$	108	-
O^1-Fe^2-S^2	107	-
Fe^1-S^1-Fe^2	74	72
Fe^1-S^2-Fe^2	75	74
S^2-Fe^3-$S_\gamma(C^{449})$	108	102
S^2-Fe^3-O^2	98	
S^2-Fe^3-$O_{\varepsilon1}(E^{556})$	96	114
$S_\gamma(C^{449})$-Fe^3-$O_{\varepsilon1}(E^{556})$	113	104
Fe^1-S^2-Fe^3	102	112
Fe^2-S^2-Fe^3	108	78
Fe^2-O^1-Fe^4	125	-
Fe^3-O^2-Fe^4	118	-
O^1-Fe^4-S^3	120	-
H_2O-Fe^4-S^3	112	-
H_2O-Fe^4-O^2	102	-
Fe^4-S^3-$S_\gamma(C^{521})$	90	-
S^3-Fe^2-$S_\gamma(C^{480})$	-	115
S^3-Fe^2-S^2	-	103
S^3-Fe^3-S^2	-	101
S^3-Fe^3-$O_{\varepsilon1}(E^{556})$	-	118
S^3-Fe^3-$S_\gamma(C^{449})$	-	118
S^3-Fe^4-$N_{\varepsilon2}(H^{259})$	-	121
S^3-Fe^4-$S_\gamma(C^{521})$	-	120
S^3-Fe^3-$O_{\varepsilon1}(E^{295})$	-	108
$N_{\varepsilon2}(H^{259})$-$Fe^4$-$S_\gamma(C^{521})$	-	103
$N_{\varepsilon2}(H^{259})$-$Fe^3$-$O_{\varepsilon1}(E^{295})$	-	101
S^1-Fe^2-S^3	-	120
Fe^3-S^3-Fe^4	-	121

Supplementary Figures

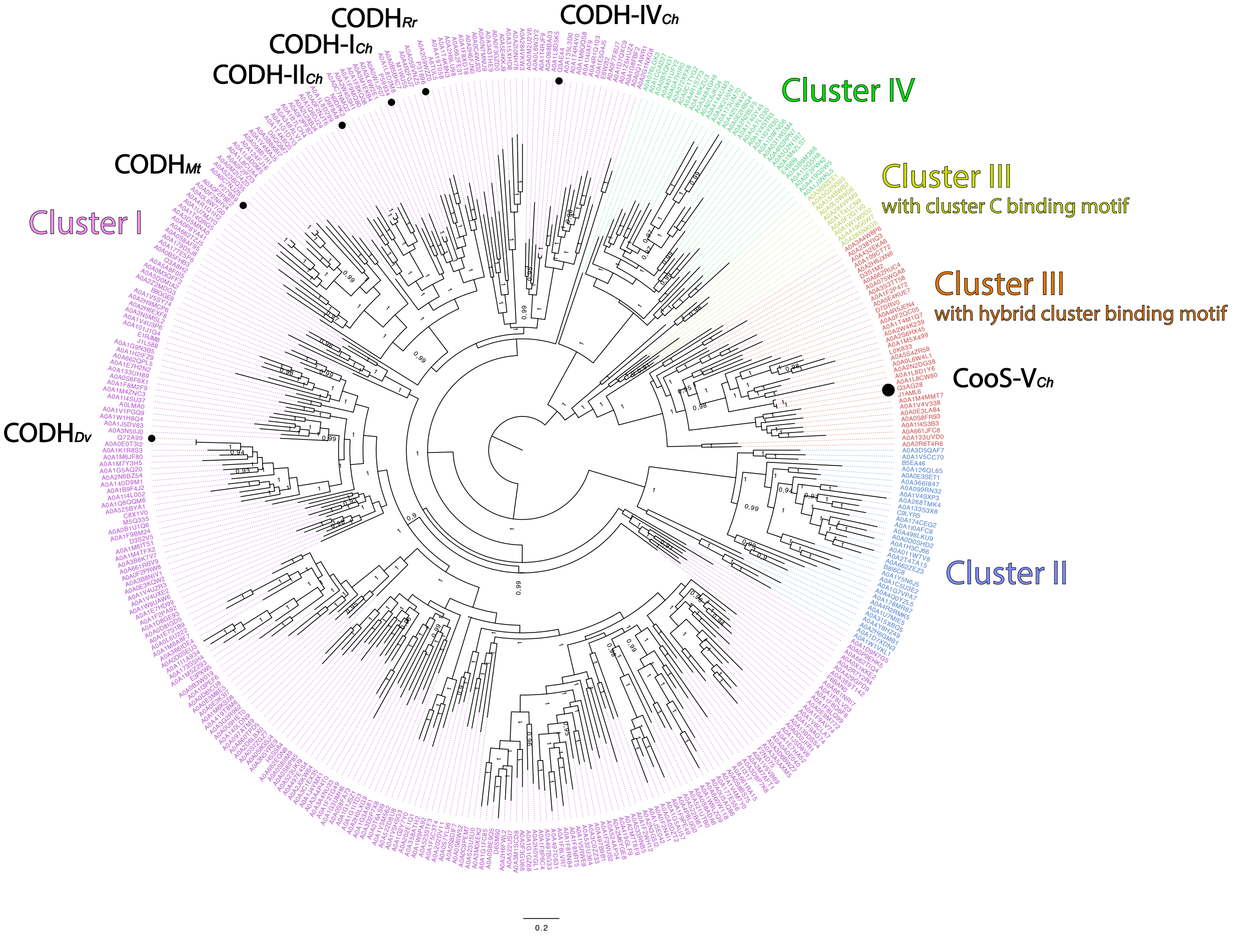

Figure S1. Phylogeny of CooS-type CODHs. Extended phylogeny of CooS-type CODHs, colored as in Figure 1. Taxa are labelled with UniProt identifiers. Branch supports are given as Bayesian-like approximate likelihood ratio test values (aBayes). Selected CODH sequences are marked by a dot with the name written next to it, where subscript indicates the source organism: Ch – *Carboxydothermus hydrogenoformans*, Dv – *Desulfovibrio vulgaris*, Mt – *Moorella thermoacetica*, Rr – *Rhodospirillum rubrum*.

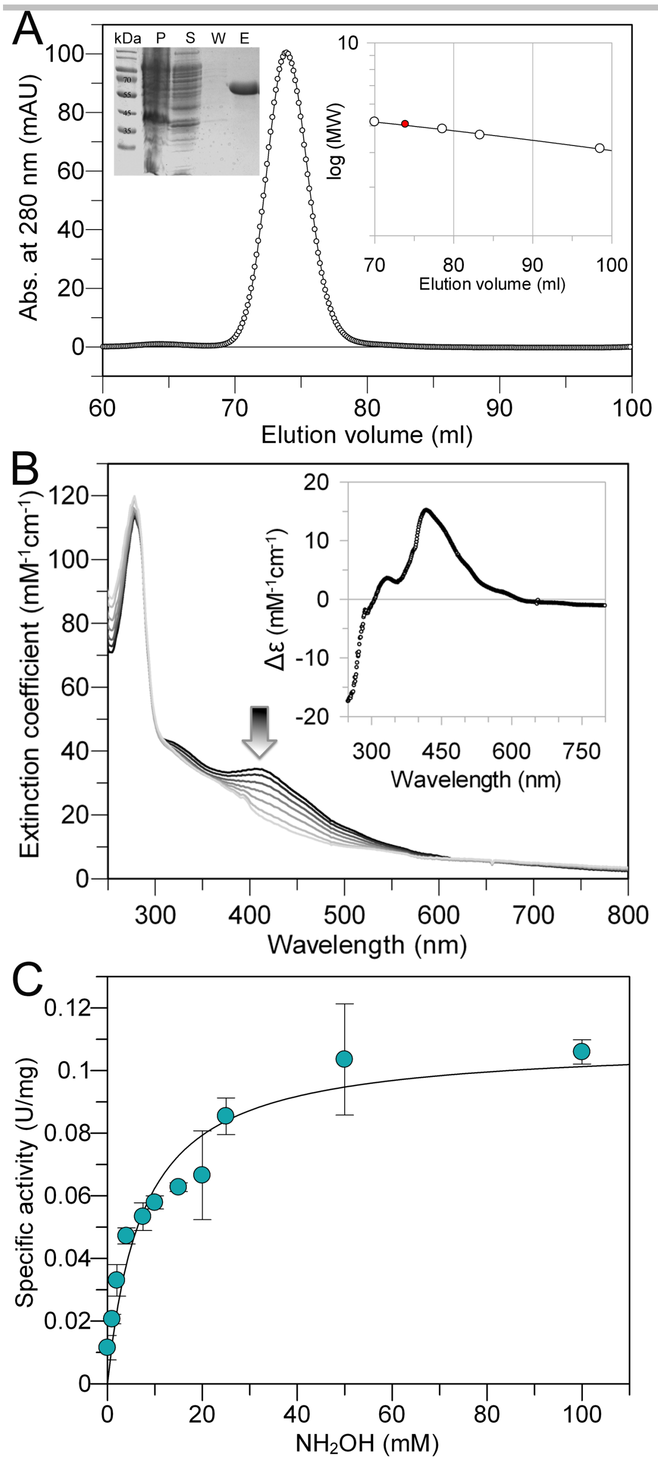

Figure S2. Biochemical characterization of CooS-V*Ch*. (A) Symmetric elution profile of CooS-V*Ch* from superdex S-200 size exclusion chromatography. In the left inset, SDS-PAGE of the purification is shown with samples: P, cell pellet; S, soluble fraction after sonication; W, washing fraction from Strep-tactin high capacity column; E, elution from the Strep-tactin column. Right inset shows elution profiles of CooS-V*Ch* with 73.9 ml elution volume (Ve, red-filled circle), corresponding to apparent molecular weight of 118 kDa that is derived from a calibration curve generated using molecular size markers (empty circles): alcohol dehydrogenase (140 kDa; Ve = 74.0 ml), albumin (66 kDa; Ve = 78.6 ml), carbonic anhydrase (29 kDa; Ve = 83.3 ml) and ribonuclease (13.7 kDa; Ve = 98.5 ml). (B) UV-vis spectroscopic reductive titration of as-isolated CooS-V*Ch*. 18 μM protein was titrated stepwise with approx. 1 μM of Na-DT in 50 mM Tris-HCl pH 8.0 with 100 mM NaCl. Bleaching of spectra is shown by color change from black to light gray (arrow). A difference spectrum (oxidized minus reduced) is shown in the inset. (C) Substrate-saturation kinetics of hydroxylamine reductase activity. The specific activity of CooS-V*Ch* was determined at different substrate concentrations by monitoring reoxidation of methyl viologen at 578 nm at 40° C. Data points were fitted by non-linear regression analysis using the Michaelis-Menten equation. Kinetic constants are determined as 7.6 ± 2.0 mM for K_M and 0.11 ± 0.01 U/mg specific activity (k_{cat} of 0.13 s^{-1}).

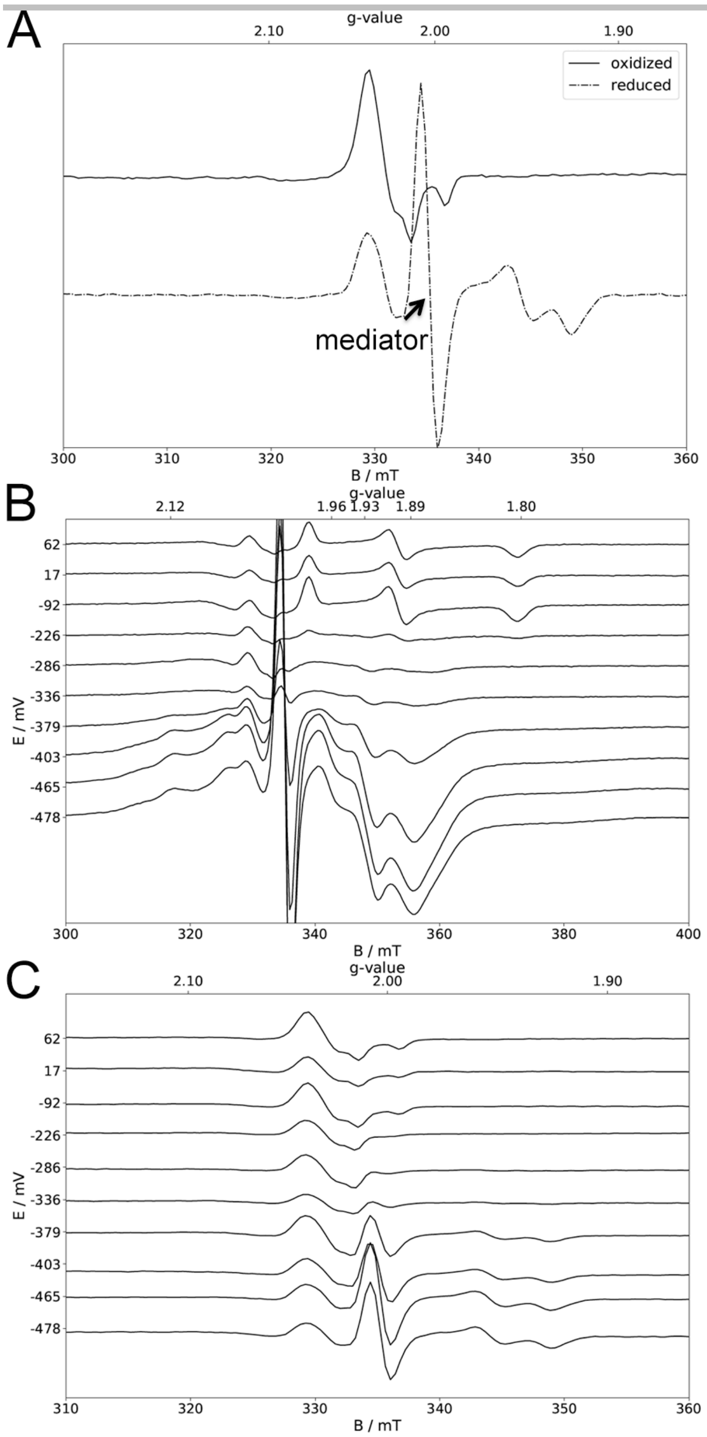

Figure S3. EPR spectroscopy. (A) EPR of CooS-V$_{Ch}$ at 80 K, the signal in the oxidized state belongs to a redox-inactive, broken Fe/S-cluster species (presumably a [3Fe-4S]-cluster), the narrow signal at g=2 stems from the added mediator mixture. f_{MW}=9.4 GHz, P_{MW}= 3mW, 10 G modulation amplitude. (B) EPR-monitored redox titration of CooS-V$_{Ch}$, T = 10 K, f_{MW}= 9.4 GHz, P_{MW}= 50 µW, 10 G modulation amplitude. (C) Redox titration of CooS-V$_{Ch}$, EPR spectra at 80 K, only the signal from the [4Fe-4S]-cluster is still visible, the spectra at -286 - +62 mV show the redox-inactive, broken Fe/S-cluster species, f_{MW}=9.4 GHz, P_{MW}= 3 mW, 10 G modulation amplitude.

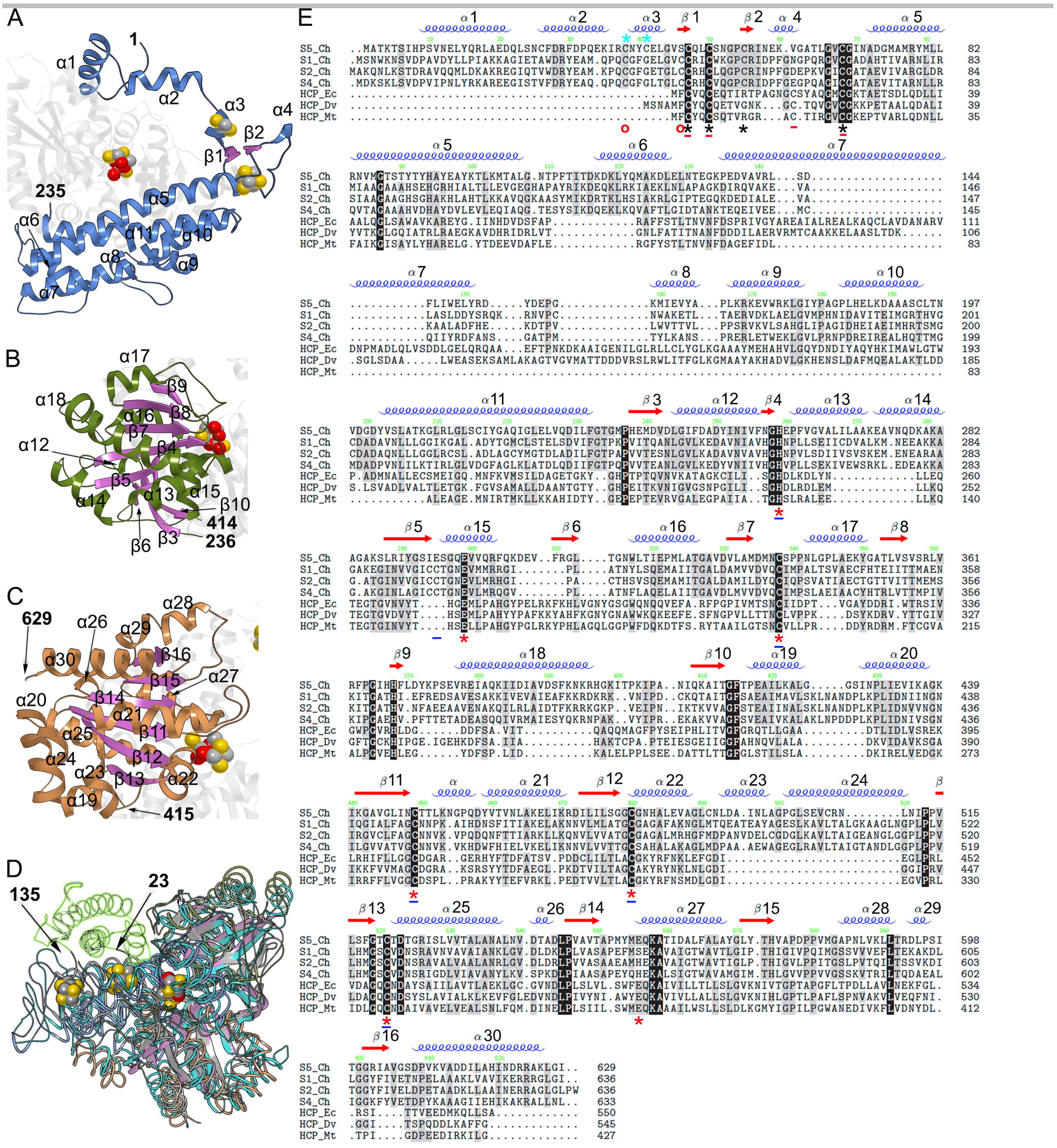

Figure S4. Topology of CooS-V$_{Ch}$. (A) N-terminal domain (1-235). (B) Middle domain (236-414). (C) C-terminal domain (415-629). In all domains, helices has same color code as domains in Fig. 2A and strands are colored in light purple. (D) Structural superposition of CooS-V$_{Ch}$ to Ni,Fe-CODH-II$_{Ch}$ (cyan, 3B53.pdb)[30] and HCP$_{Dv}$ (gray, 1OA1.pdb)[13]. Structurally deviating N-terminal residues 23-135 from HCP are indicated (green outline). (E) Sequence alignment. Helices and strands are numbered as in A-C. Stars indicate residues for the hybrid-clusters in CooS-V$_{Ch}$ (cyan for [2Fe-2S]-cluster, black for [4Fe-4S]-cluster and red for the hybrid-cluster). Red circles indicate Cys residues coordinating cluster D in Ni,Fe-CODH. Blue bars indicate residues coordinating cluster C in Ni,Fe-CODH and red bars residues coordinating the [4Fe-4S]-cluster in HCP. Abbreviations: Ec, *Escherichia coli*; Dv, *Desulfovibrio vulgaris*; Mt, *Methanobacterium thermoautotrophicum*.

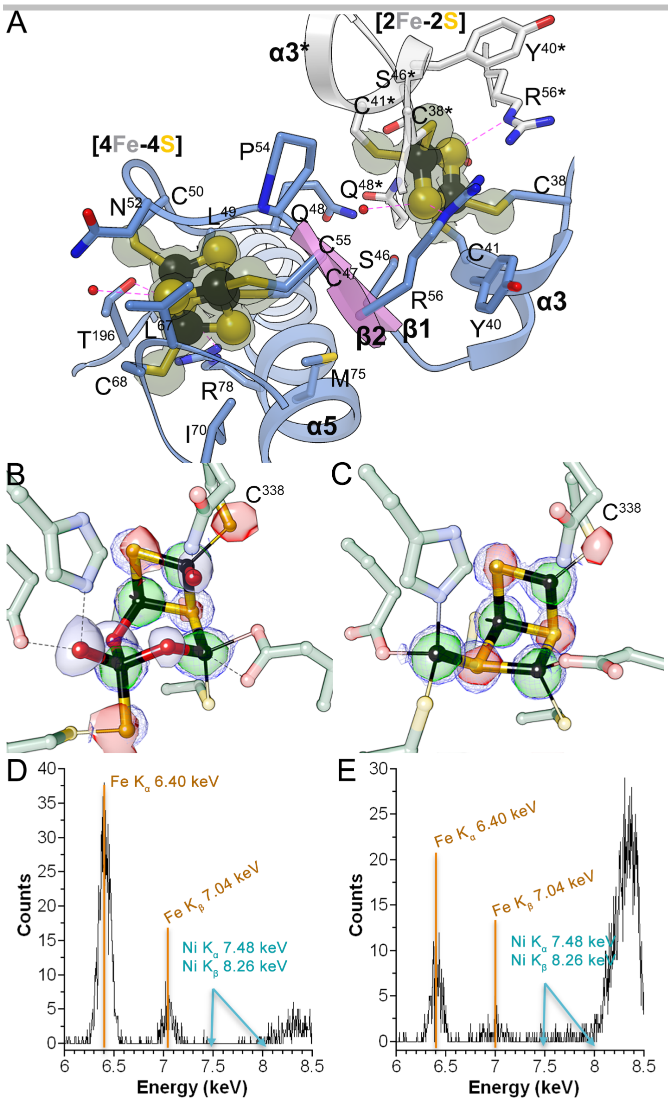

Figure S5. Electron density representation. (A) Environment around the [2Fe-2S]-cluster and [4Fe-4S]-cluster. A sigma-A weighted 2F_o-F_c map is shown as gray surface with 3 rmsd Pink dashes indicate H-bonding distance within 3.2 Å. Stars represent a symmetric molecule. Same color code as in Fig. 3 for domains. Electron density maps for oxidized (B) and reduced (C) structures. Anomalous difference densities for iron (green, 10 rmsd, λ = 1.74 Å) and sulfur (red, 4 rmsd, λ = 1.90 Å) atoms are shown as surface. Sigma-A weighted F_o-F_c omit electron densities without modeling of the cluster are shown in blue surface at 4 rmsd. For comparison, the anomalous scattering peak from Sγ of C338 is included. X-ray fluorescence spectra of CooS-V samples in crystal (D) and solution (310 μM) (E).

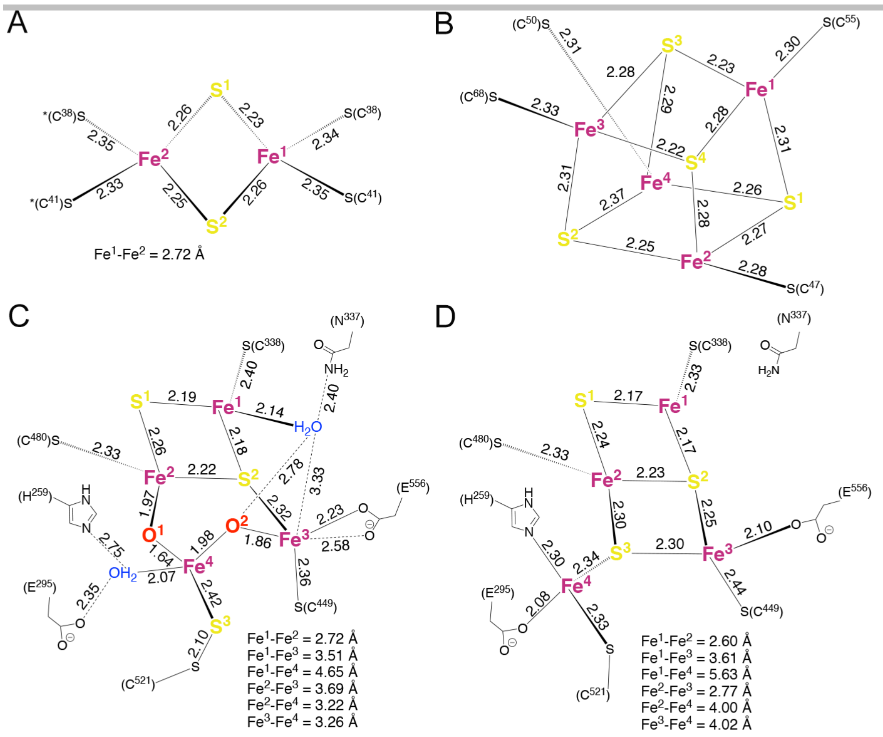

Figure S6. Schematic drawing of metal clusters. (A) [2Fe-2S]-cluster. (B) [4Fe-4S]-cluster. (C) Oxidized hybrid-cluster. (D) Reduced hybrid-cluster. Bond lengths in (A) and (B) are for the oxidized state, which are practically unchanged in the reduced structure. Bond lengths are shown in Angstrom. Given distances are average values from two molecules in asymmetric unit, except for (A).

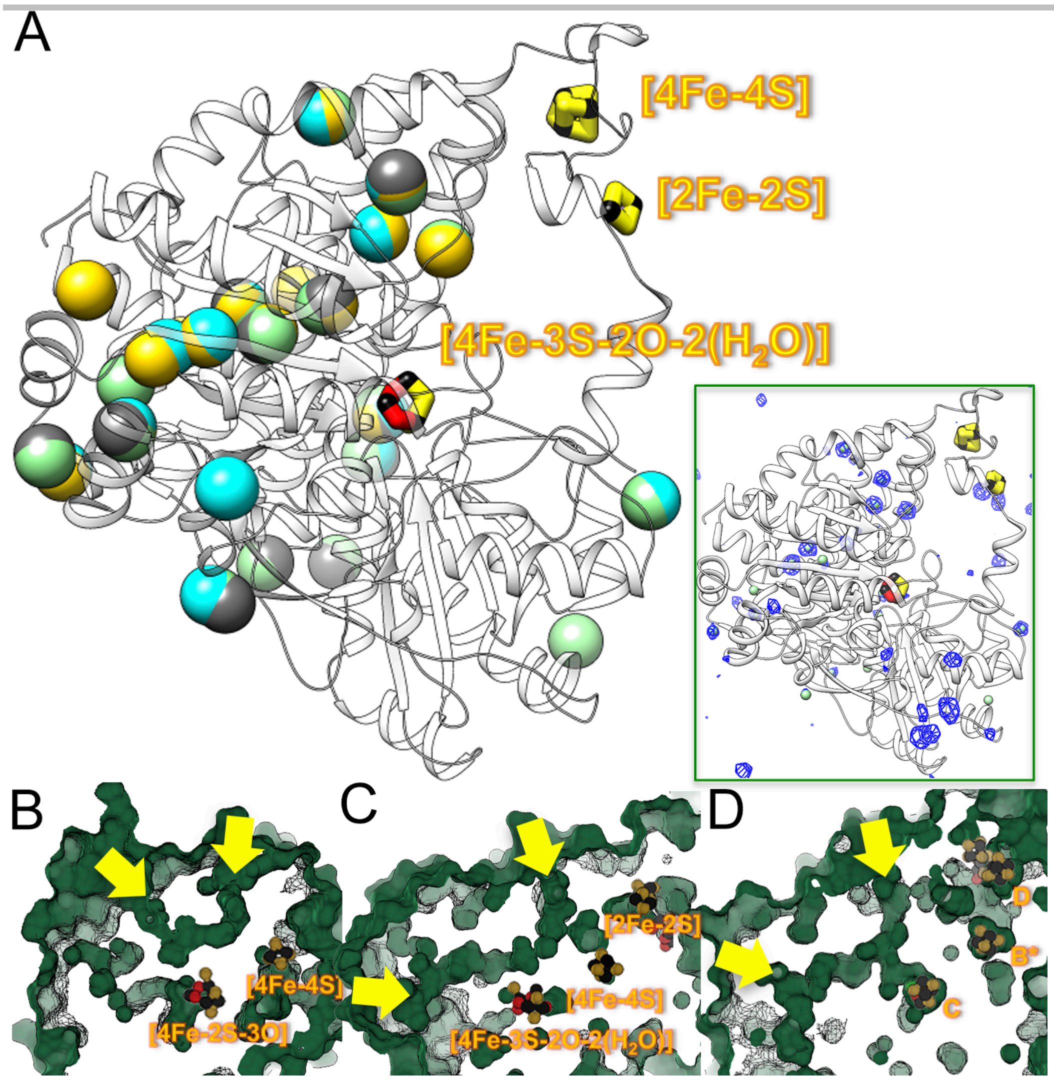

Figure S7. Analysis of channels. (A) Xe-derivatized structure of CooS-V*Ch*. Xe atoms (spheres) found in four molecules of the ASU are superposed and shown in different colors (gold, gray, cyan, and green). Only one molecule of CooS-V*Ch* is shown as white cartoon. Metal-clusters are shown as sticks. The model is similarly oriented as C for comparison. Anomalous difference electron density for Xe is shown in the green box with 3.5 rmsd (blue mesh). Channel comparison of (B) HCP (pdb-ID:1OA1.pdb[31]); (C) oxidized CooS-V*Ch* and (D) CODH-II*Ch* (pdb ID: 3B5B.pdb[32]). All structures (B-C) are superposed and shown in the same orientation.

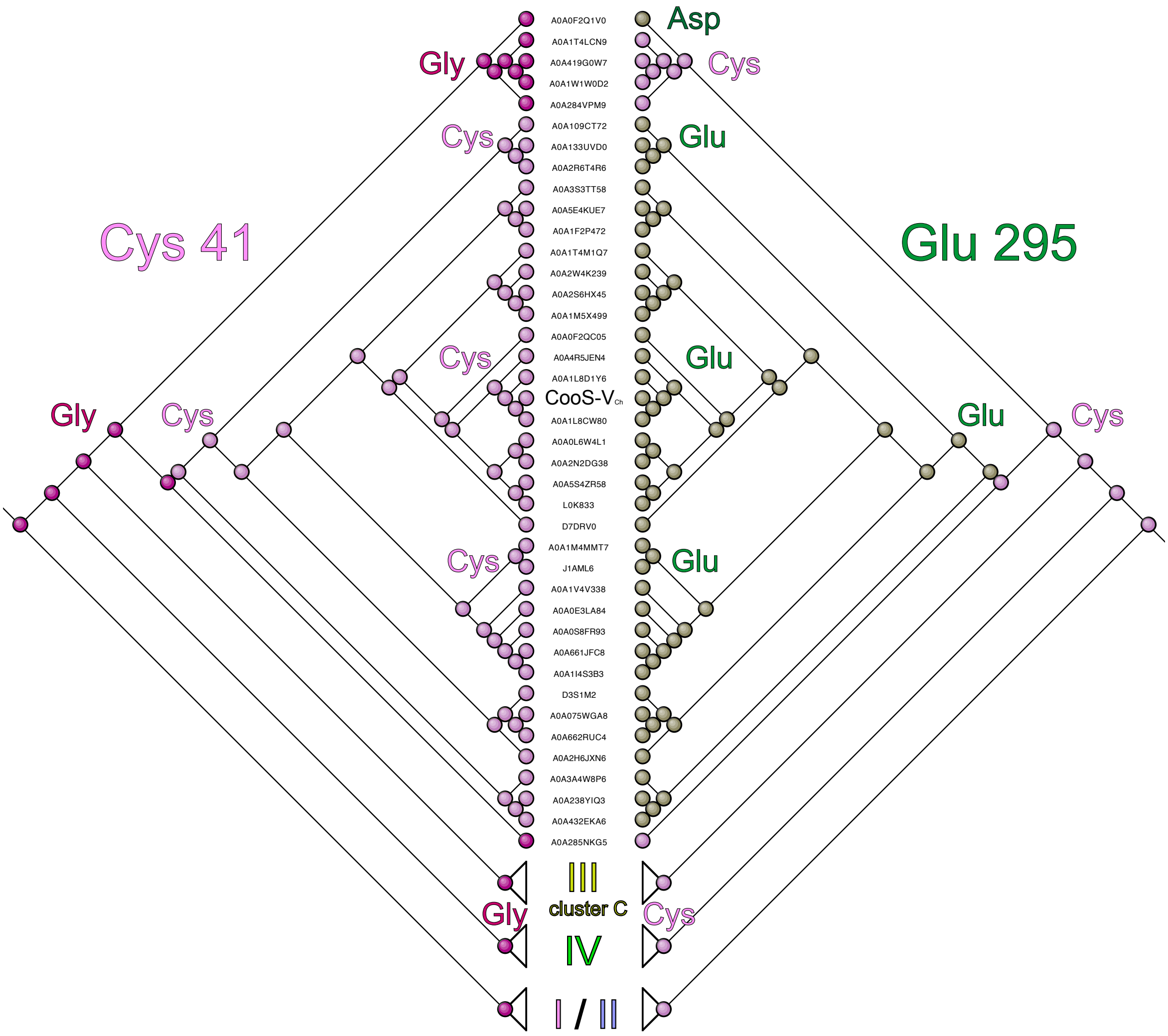

Figure S8. Mirror tree cladogram for the history of Cys41 and Glu295. Selected part of the phylogeny shown in Figure S1 with character history for the sequence positions corresponding to Cys41 (characteristic for the presence of the [2Fe-2S]-cluster at cluster D) and Glu295 (exchange of cluster C coordinating Cys for Glu, characteristic for the binding motif of the hybrid-cluster). Colored balls indicate the type of amino acid present in the sequence (for terminal taxa) and most parsimonious amino acid (for ancestral nodes). Terminal nodes for sequences of sequence clusters I, II, III (cluster C coordinating motif) and IV were merged. CooS-V$_{Ch}$ is indicated, other terminal taxa give UniProt Ids.

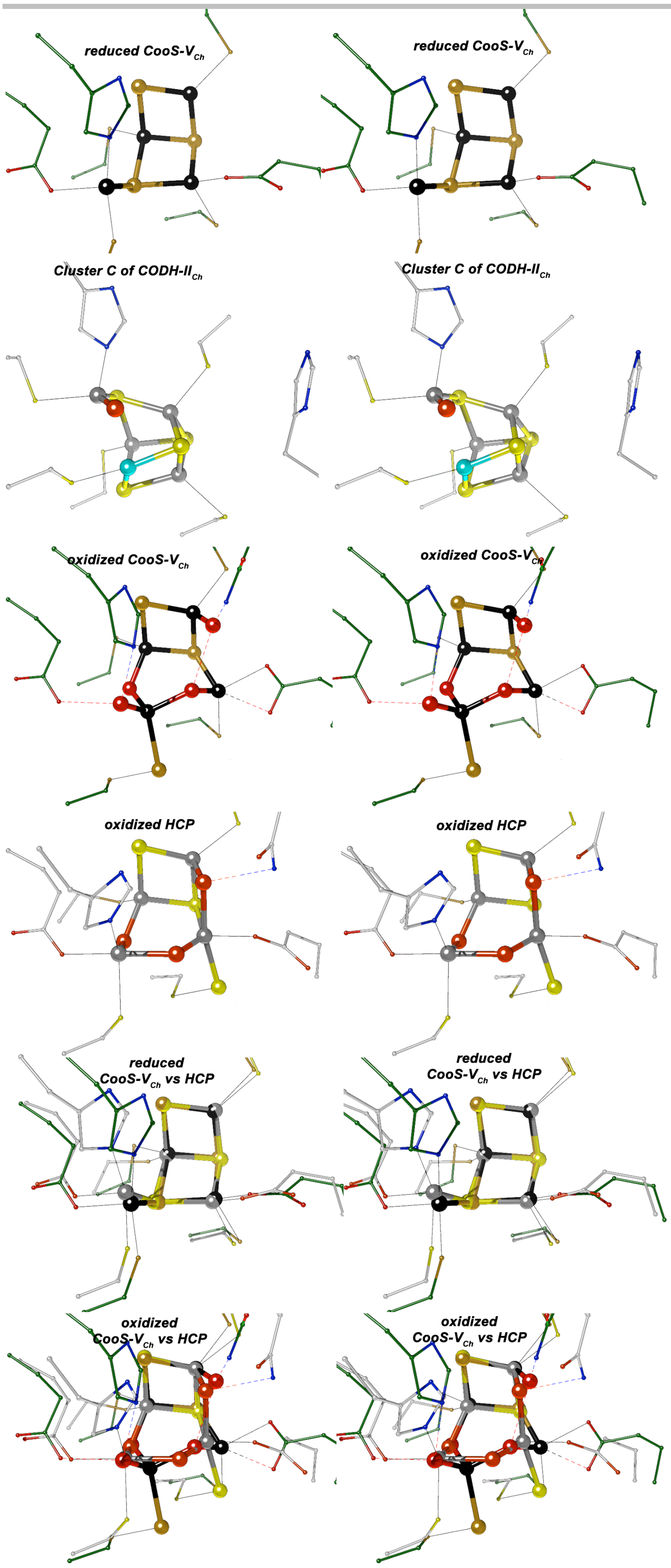

Figure S9. Stereo view of Figure 7.

References

[1] A. L. Mitchell, T. K. Attwood, P. C. Babbitt, M. Blum, P. Bork, A. Bridge, S. D. Brown, H.-Y. Chang, S. El-Gebali, M. I. Fraser, J. Gough, D. R. Haft, H. Huang, I. Letunic, I. Letunic, R. Lopez, A. Luciani, F. Madeira, A. Marchler-Bauer, H. Mi, D. A. Natale, M. Necci, G. Nuka, C. Orengo, A. P. Pandurangan, T. Paysan-Lafosse, S. Pesseat, S. C. Potter, M. A. Qureshi, N. D. Rawlings, N. Redaschi, L. J. Richardson, C. Rivoire, G. A. Sutton, N. Thanki, P. D. Thomas, S. C. E. Tosatto, S.-Y. Yong, R. D. Finn, *Nucleic Acids Res.* **2019**, *47*, D351–D360.

[2] H. J. Atkinson, J. H. Morris, T. E. Ferrin, P. C. Babbitt, *PLoS ONE* **2009**, *4*, e4345–.

[3] P. Shannon, A. Markiel, O. Ozier, N. S. Baliga, J. T. Wang, D. Ramage, N. Amin, B. Schwikowski, T. Ideker, *Genome Res.* **2003**, *13*, 2498–2504.

[4] K. Katoh, D. M. Standley, *Mol. Biol. Evol.* **2013**, *30*, 772–780.

[5] D. Darriba, G. L. Taboada, R. Doallo, D. Posada, *Bioinformatics* **2011**, *27*, 1164–1165.

[6] S. Guindon, J. F. Dufayard, V. Lefort, M. Anisimova, W. Hordijk, O. Gascuel, *Syst. Biol.* **2010**, *59*, 307–321.

[7] S. Q. Le, O. Gascuel, *Mol. Biol. Evol.* **2008**, *25*, 1307–1320.

[8] M. Anisimova, M. Gil, J. F. Dufayard, C. Dessimoz, O. Gascuel, *Syst Biol* **2011**, *60*, 685–699.

[9] F. D. K. Tria, G. Landan, T. Dagan, *Nat. Ecol. Evol.* **2017**, *1*, 193.

[10] Z. Yang, *Bioinformatics* **1997**, *13*, 555–556.

[11] W. P. Maddison, D. R. Maddison, “Mesquite: A modular system for evolutionary analysis, version 3.61,” can be found under http://www.mesquiteproject.org, **2019**.

[12] A. Rambaut, Computer program distributed by the author, website: *http://tree.bio.ed.ac.uk/software/figtree*. **2020**.

[13] L. Pravda, D. Sehnal, D. Toušek, V. Navrátilová, V. Bazgier, K. Berka, R. S. Vařeková, J. Koča, M. Otyepka, *Nucleic Acids Res.* **2018**, *46*, W368–W373.

[14] E. F. Pettersen, T. D. Goddard, C. C. Huang, G. S. Couch, D. M. Greenblatt, E. C. Meng, T. E. Ferrin, *J. Comput. Chem.* **2004**, *25*, 1605–1612.

[15] M. Wu, Q. Ren, A. S. Durkin, S. C. Daugherty, L. M. Brinkac, R. J. Dodson, R. Madupu, S. A. Sullivan, J. F. Kolonay, W. C. Nelson, L. J. Tallon, K. M. Jones, L. E. Ulrich, J. M. Gonzalez, I. B. Zhulin, F. T. Robb, J. A. Eisen, *PLoS Genet.* **2005**, *1*, e65.

[16] J.-H. Jeoung, H. Dobbek, *Science* **2007**, *318*, 1461–1464.

[17] W. W. Fish, in *Metallobiochemistry Part A*, Elsevier, **1988**, pp. 357–364.

[18] M. M. Bradford, *Anal. Biochem.***1976**, *72*, 248–254.

[19] M. T. Wolfe, J. Heo, J. S. Garavelli, P. W. Ludden, *J. Bacteriol.* **2002**, *184*, 5898–5902.

[20] R. J. Leatherbarrow, *Erithacus Software Ltd., Horley, U.K.* **2009**.

[21] F. H. Thorndycroft, G. Butland, D. J. Richardson, N. J. Watmough, *Biochem. J.* **2007**, *401*, 111–119.

[22] F. W. Studier, *Protein Expr. Purif.* **2005**, *41*, 207–234.

[23] J.-H. Jeoung, H. Dobbek, *Proc. Natl. Acad. Sci. U.S.A.* **2018**, *115*, 2994–2999.

[24] U. Mueller, R. Förster, M. Hellmig, F. U. Huschmann, A. Kastner, P. Malecki, S. Pühringer, M. Röwer, K. Sparta, M. Steffien, M. Ühlein, P. Wilk, M. S. Weiss, *Eur. Phys. J. Plus* **2015**, *130*, 141.

[25] M. Krug, M. S. Weiss, U. Heinemann, U. Mueller, *J. Appl. Crystallogr.* **2012**, *45*, 568–572.

[26] P. D. Adams, P. V. Afonine, G. Bunkóczi, V. B. Chen, I. W. Davis, N. Echols, J. J. Headd, L. W. Hung, G. J. Kapral, R. W. Grosse-Kunstleve, A. J. McCoy, N. W. Moriarty, R. Oeffner, R. J. Read, D. C. Richardson, J. S. Richardson, T. C. Terwilliger, P. H. Zwart, *Acta Crystallogr. D Biol. Crystallogr.* **2010**, *66*, 213–221.

[27] J. Heo, M. T. Wolfe, C. R. Staples, P. W. Ludden, *J. Bacteriol.* **2002**, *184*, 5894–5897.

[28] P. Emsley, B. Lohkamp, W. G. Scott, K. Cowtan, *Acta Crystallogr. D Biol. Crystallogr.* **2010**, *66*, 486–501.

[29] L. Holm, L. M. Laakso, *Nucleic Acids Research* **2016**, *44*, W351–W355.

[30] S. Macedo, D. Aragão, E. P. Mitchell, P. Lindley, *Acta Crystallogr. D Biol. Crystallogr.* **2003**, *59*, 2065–2071.

[31] D. Aragão, S. Macedo, E. P. Mitchell, C. V. Romão, M. Y. Liu, C. Frazão, L. M. Saraiva, A. V. Xavier, J. LeGall, W. M. A. M. van Dongen, W. R. Hagen, M. Teixeira, M. A. Carrondo, P. Lindley, *J. Biol. Inorg. Chem.* **2003**, *8*, 540–548.

[32] H. Dobbek, V. Svetlitchnyi, L. Gremer, R. Huber, O. Meyer, *Science* **2001**, *293*, 1281–1285.

Author Contributions

LD and FK prepared and crystallized proteins. JF performed biochemical characterizations and solved the first crystal structure. MS performed EPR experiments. CT performed and analyzed EPR experiments and wrote the paper. J.-H.J. initiated and designed the study, treated crystals, collected diffraction data, determined the crystal structures, analyzed data and wrote the paper. HD designed the study, analyzed sequence data and wrote the paper.

All authors have given approval to the final version of the manuscript.
